# Hunter-Gatherers Harvested and Heated Microbial Biogenic Iron Oxides to Produce Rock Art Pigment

**DOI:** 10.1038/s41598-019-53564-w

**Published:** 2019-11-19

**Authors:** Brandi Lee MacDonald, David Stalla, Xiaoqing He, Farid Rahemtulla, David Emerson, Paul A. Dube, Matthew R. Maschmann, Catherine E. Klesner, Tommi A. White

**Affiliations:** 1 Archaeometry Laboratory, University of Missouri Research Reactor, Columbia, MO 65211 USA; 2 Electron Microscopy Core, University of Missouri, Columbia, MO 65211 USA; 3 Department of Anthropology, University of Northern British Columbia, Prince George, BC V2N4Z9 Canada; 4 Bigelow Laboratory for Ocean Sciences, East Boothbay, ME 04544 USA; 5 Brockhouse Institute for Materials Research, McMaster University, Hamilton, L8S4M1 Canada; 6 Mechanical and Aerospace Engineering, University of Missouri, Columbia, MO 65211 USA; 7 Department of Materials Science and Engineering, University of Arizona, Tucson, AZ 87521 USA; 8 Biochemistry, University of Missouri, Columbia, MO 65211 USA

**Keywords:** Environmental social sciences, Biochemistry

## Abstract

Red mineral pigment use is recognized as a fundamental component of a series of traits associated with human evolutionary development, social interaction, and behavioral complexity. Iron-enriched mineral deposits have been collected and prepared as pigment for use in rock art, personal adornment, and mortuary practices for millennia, yet little is known about early developments in mineral processing techniques in North America. Microanalysis of rock art pigments from the North American Pacific Northwest reveals a sophisticated use of iron oxide produced by the biomineralizing bacterium *Leptothrix ochracea;* a keystone species of chemolithotroph recognized in recent advances in the development of thermostable, colorfast biomaterial pigments. Here we show evidence for human engagement with this bacterium, including nanostructural and magnetic properties evident of thermal enhancement, indicating that controlled use of pyrotechnology was a key feature of how biogenic iron oxides were prepared into paint. Our results demonstrate that hunter-gatherers in this area of study prepared pigments by harvesting aquatic microbial iron mats dominated by iron-oxidizing bacteria, which were subsequently heated in large open hearths at a controlled range of 750 °C to 850 °C. This technical gesture was performed to enhance color properties, and increase colorfastness and resistance to degradation. This skilled production of highly thermostable and long-lasting rock art paint represents a specialized technological innovation. Our results contribute to a growing body of knowledge on historical-ecological resource use practices in the Pacific Northwest during the Late Holocene.

Figshare link to figures: https://figshare.com/s/9392a0081632c20e9484.

## Introduction

Red pigments, broadly referred to as ochre, are the most commonly identified mineral pigments used throughout human history worldwide^1–9^. Ochre use is a typical characteristic of North America’s earliest Paleoindian inhabitants^10–12^, although whether considered ritual or utilitarian remains a longstanding debate^13–15^. In Beringia and the Pacific Northwest, its use is documented as early as 11.5 kya^16^, and is sustained through to the European contact era as a component of ceremonial and symbolic practices, rock art painting, and socioeconomic interaction in hunter-gatherer communities^17–22^. Despite its widespread use, little attention has been paid to ochre technologies in North America, including mineral collection, preparation, and thermal treatment, as characteristic features of the pigment production *chaîne opératoire*. These steps, including levigation, homogenization, blending with opacifiers or binders, were fundamental to achieve the desired consistency, texture, and hue of ochre paint. Those preparatory steps are infused with detailed information on the skilled knowledge of the pigment producers. We focus here on a late Holocene Pacific Northwest example, yet this study bears broader relevance for reconstructing key evidence for pyrotechnological innovations and complex cognitive processes.

At a regional scale, clusters of rock art (pictographs and petroglyphs), dot the landscape of the Pacific Northwest. Despite a long and demonstrated history of ochre use, mineral pigment preparation for pictographic rock art in this area is poorly understood. Rock art research has traditionally focused on interpreting glyphs and imagery^17,23^, with lesser emphasis on the paints and how they were produced, although see Wainwright^24^. They are described by descendant communities and in ethnohistoric records as important communication tools for stories about spiritual beings, hunting grounds, and places and events of historical significance^25–27^. Our study focuses on paint used to produce a pictograph panel at Boling Point (GcSi-1), located on the south arm of Babine Lake in the central Interior Plateau region of British Columbia (Canada). While the temporal sequence of rock art production of Babine Lake is unknown, human occupation in the region dates as early as 5,000 years BP^28^. Across the lake there are over 150 individual monochrome (red) glyphs, all of which are painted on prominent, open-air rock faces. The imagery is comprised of figurative anthropomorphic, animal (otter, fish, cariboo, beaver, toad, bear, bird, snake), object (canoe, sticks, fish weirs, various tools) and geometric figures (circles, lines, chevrons, diamonds, tally marks)^27^ (see SI Fig. 1a–h for examples). In ethnographic records, Morice^27^ describes some images as short communications between mobile hunting parties concerning ideal camping sites or hunting or fishing areas, distances between campsites, potential hazards, the injury or death of a party member, or the presence of other parties passing through the area. In some instances, they were drawn using charcoal and not intended to have a long-term presence on the landscape. Charcoal drawings are no longer readily visible, but most figures drawn using red ochre pigment remain observable to present day. Very few records exist, oral historical or written, that describe any aspects of pigment procurement or production in this area. However, one potential source of biogenic iron oxide for paint production was identified by B.L.M. and F.R. during recent field survey and will be examined in future studies.

Babine Lake is situated equidistant to the Skeena (which it flows into) and Fraser Rivers, two major waterways that run through north interior British Columbia. As such, Babine Lake is a historically-significant node for regional scale socioeconomic interaction. This nexus connected Pacific Coastal communities to the Interior Plateau by way southwest to the Bella Coola Valley (Nuxalk, Heiltsuk), south and east to Plateau and Fraser Valley communities, north through extensive trail systems (including to the key obsidian source at Mt. Edziza^29^), and north-west along the Skeena River to Tsimshian-speaking groups on the North Coast^18^. Ethnohistoric documentation lists red ochre pigment as a common exchange item between Coastal and Interior communities along this route^30^. Despite the inherent value of oral historical and ethnographic evidence, our technological questions regarding the selection, preparation, and application of ochre mineral pigments remain unanswered.

**Figure 1:**
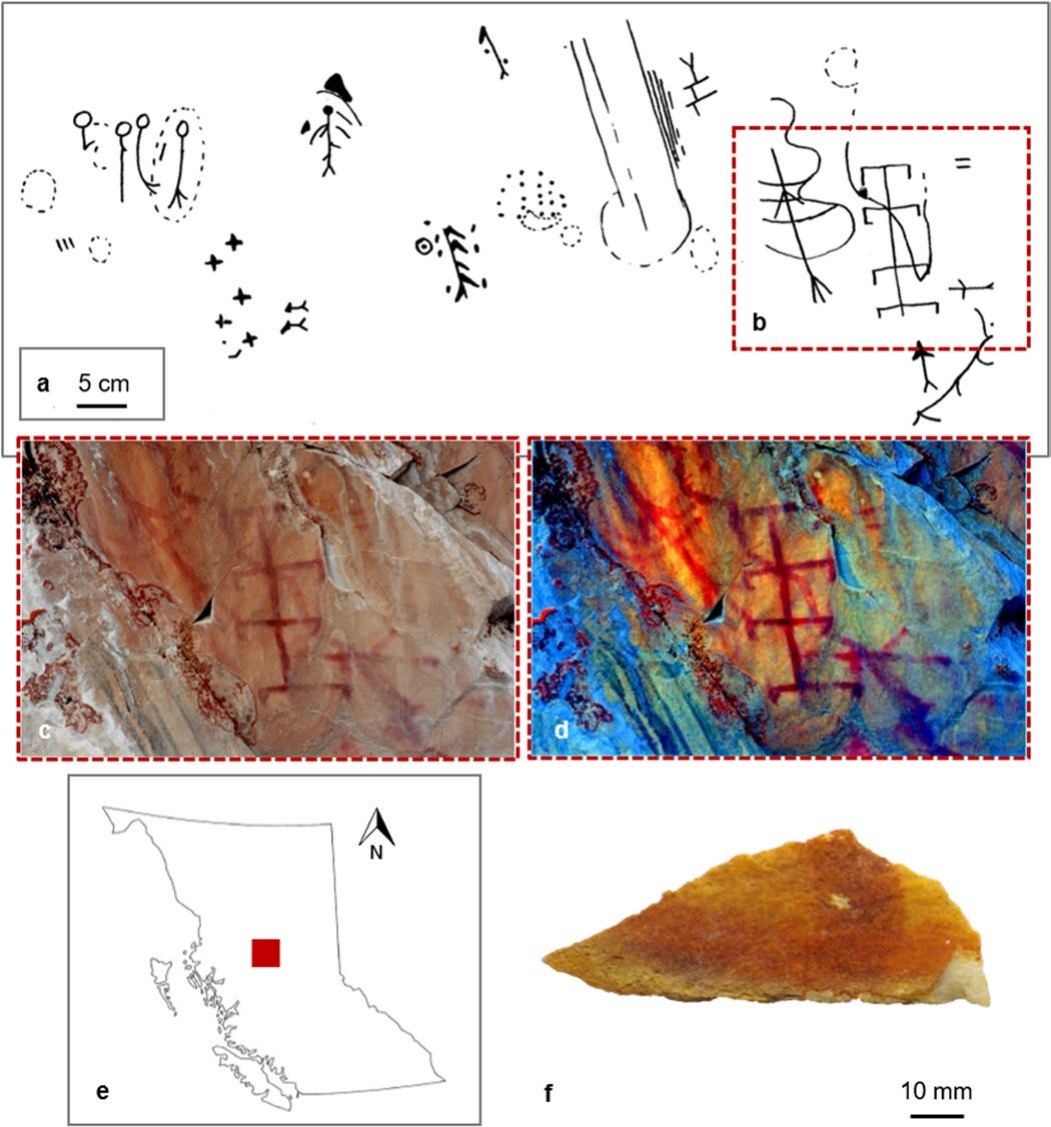
Context of Boling Point and Babine Lake Rock Art. (**a**) Sketch drawings of section of Boling Point pictograph, adapted from Mohs and Mohs^84^; (**b**) area of rock art panel shown in photographs and where sample was taken; (**c,d**) photograph of Boling Point rock art panel in color (**c**) and digitally enhanced with D-Stretch (**d**), Babine Lake (courtesy of G. Keddie, Royal BC Museum); (**e**) map of British Columbia, highlighting area of study; (**f**) extracted fragment of Boling Point pictograph from Mohs and Mohs^84^ survey, from the Royal British Columbia Museum archive (Vancouver, Canada).

We present here two important findings. Multiple independent lines of evidence unite to show that the individuals who prepared paints for rock art at Babine Lake harvested aquatic microbial iron mats dominated by iron-oxidizing bacteria (FeOB) (Fig. 1, SI Text 1). Those bacterial species produce biominerals with unique morphologies that can be long-lived^31^. This iron-rich material was homogenized and heated in large domestic hearths at a controlled range of approximately 750 °C to 850 °C; a technical gesture that was deliberately performed to enhance color properties, transforming orange-brown sediment to a vivid red hue. The heat treatment process converted the non-crystalline iron oxide minerals to crystalline forms resulting in increased colorfastness and resistance to degradation. This selective production of durable, colorfast, and highly thermostable biogenically-derived rock art paint represents a unique technological innovation. Our findings contribute to a growing body of literature on how hunter-gatherer communities in the Pacific Northwest possessed skilled ecological knowledge^32^. We also advance knowledge on the nanostructure, thermostability, and diagenesis of *L*. *ochracea* biomineral nanocomposites, with implications for the contemporary commercial production of renewable, thermostable, colorfast red pigments^33,34^.

## Reconstructing Ochre Pigment Technology

The term ochre widely denotes red pigmentaceous material, yet encompasses a range of rocks and minerals bearing iron oxides/oxyhydroxides capable of producing a streak or stain, including ferrihydrite, hematite (Fe_2_O_3_), goethite (FeOOH), magnetite (Fe_3_O_4_) and limonite (FeO·nH_2_O). Differences in ochre pigments are not always readily identifiable, and pigments that appear similar in color and texture can have distinct elemental, mineralogical, and physical properties^35^. This recent recognition has ushered a surge of interest in characterizing and reconstructing aspects of ochre pigment treatment, such as raw material selection^36–39^, mineral processing technologies^40–43^, paint recipes (organic binders, inorganic adjuncts)^44–46^, and thermal enhancement^47–49^. However, a type of iron oxide source that is ecologically abundant yet relatively understudied is that which is formed by aquatic iron oxide producing bacteria (FeOB).

**Figure 2:**
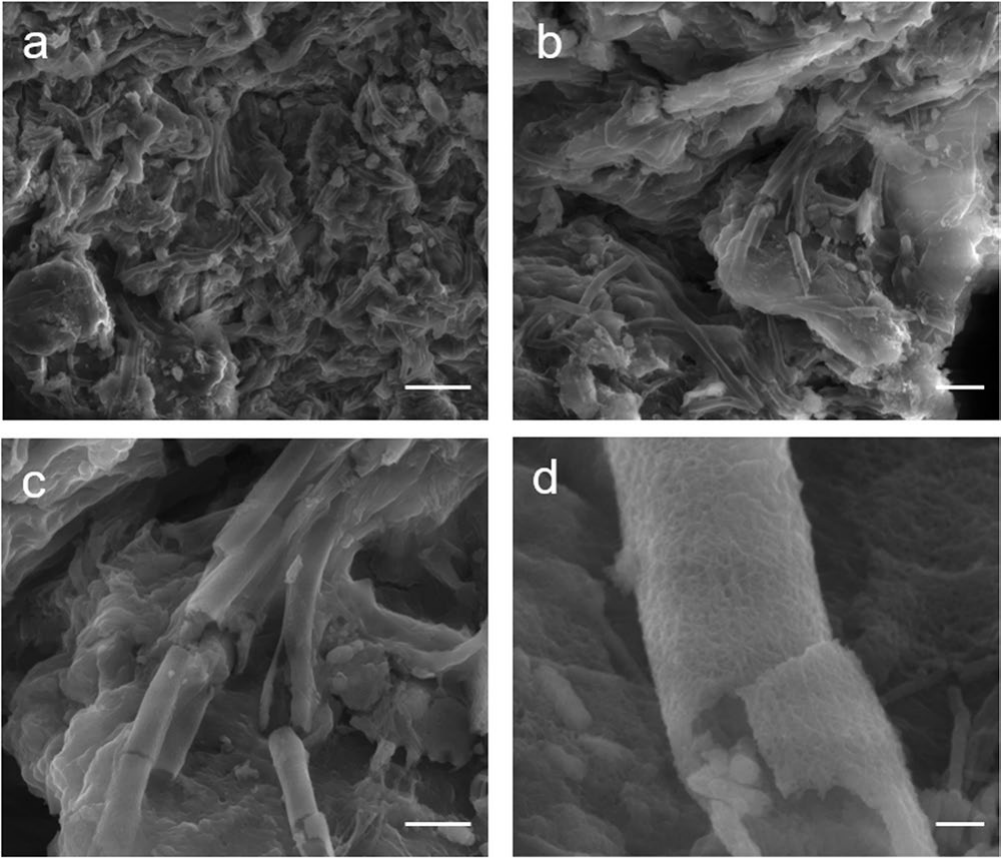
Structure and morphology of a *L*. *ochracea* microcolony cluster on a sediment particle. Scanning electron micrographs using secondary electrons illustrating freshly collected and untreated *L*. *ochracea*, collected by D. Emerson. (**a**) Large cluster of sheaths on a sediment particle. Scale = 10 µm. (**b**) Cluster of *L*. *ochracea* growth. Scale = 5 µm. (**c**) Detail of *L*. *ochracea* cluster showing unidirectional growth pattern. Note the relatively smooth surface texture. Scale = 2.5 µm. (**d**) Detail of broken *L*. *ochracea* sheath. Scale = 0.25 µm.

FeOB are chemosynthetic microbes that gain energy through the oxidation of iron, and play an important role in the biogeochemical cycling of iron^50^. They typically grow in aquatic habitats, such as wetlands or creek banks, where ferrous iron-rich groundwater mingles with oxygenated surface waters, and produce visible, rust-colored microbial mats that can cover tens of square meters (SI Text 2). Microscopic analysis of these mats reveals a variety of morphotypes of biomineral precipitates produced by the growth of the bacteria^31^. *L*. *ochracea* is often ubiquitous in these mats. It excretes a hollow, tubular sheath that is typically a micron in diameter, but can be hundreds of microns in length. The sheath is primarily composed of poorly crystalline iron-oxides organized as a fine fibrillar matrix with an organic component^51–53^. The resulting biomineral is a highly thermostable iron-silicate nanocomposite of Fe:Si:P, plus structural O, C, and H^54^ (Fig. 2). Consistent with this stability, fossilized remnants of *L*. *ochracea* sheaths are preserved in the rock record and provide evidence for the presence of FeOB in ancient samples^55,56^. In present day commercial paint production, red pigments derived from *L*. *ochracea* are touted as renewable, colorfast, non-toxic, and highly thermostable biomaterials^33,34^, all likely the same characteristics that made them desirable to people in the prehistoric past.

The challenge for reconstructing pyrotechnological behaviors is developing suitable proxies for modelling those activities, and little is known about how thermal treatment of ochre developed over time. In abiotic iron oxides, moderate to high temperature thermal exposure (200 °C to 500 °C) will accelerate oxidation, induce phase change, and transform color properties^57^. The visible outcome is a transition from brown, yellow, or orange to increasingly vivid red and red-purple hues, followed by grey or black. Those transformations, typically achieved through thermal alteration of limonite or goethite to hematite, magnetite, and maghemite (γ-Fe_2_O_3_), can vary depending on peak temperature, redox conditions, organic matter content^58^, heating environment, particle size^59^, and surface area^60–63^, but generally occur below 500 °C. However, *L*. *ochracea* iron-silicate biominerals are highly thermostable, requiring markedly greater temperatures (700 °C+) to induce these changes and achieve color enhancement^33^. Therefore, traditional models for thermally-induced transformations in abiotic iron oxides cannot be used to model the processes that occur during the heating of *L*. *ochracea* biominerals. Accordingly, our interrelated aim was to develop a model for reconstructing how biogenic iron oxides were thermally enhanced and prepared in antiquity.

## Results

### Experimental design

At the outset of this study our objective was to characterize the pigment used to produce rock paintings at Babine Lake using a combination of scanning electron microscopy (SEM), micro-Raman spectroscopy, and X-ray diffraction (XRD). Initial investigation showed that the pigment admixture was composed of microfossil sheaths of *L*. *ochracea*, whose features potentially indicated morphological and nanostructural evidence for thermal enhancement. That unexpected discovery was the impetus for a concurrent control study in which we subjected freshly collected specimens of *L*. *ochracea* (see SI Text 2) to a series of comparative analyses, focusing on methods to evaluate the potential for their heat treatment in the past. Six aliquots of *L*. *ochracea* control samples underwent two heating procedures: (a) by muffle furnace at increasing dwell temperature (200 °C, 400 °C, 600 °C, 800 °C, and 1,000 °C), and; (b) by continuous *in situ* SEM heating. Subsequently, the Babine Lake rock art fragment (hereafter referred to as GcSi-1) and FeOB control samples were analyzed by electron microscopy/microanalysis methods (SEM, transmission electron microscopy or TEM, X-ray energy dispersive spectroscopy or EDS), XRD, and SQUID (superconducting quantum interference device) magnetometry.

### Babine Lake rock art and microenvironment

**Figure 3:**
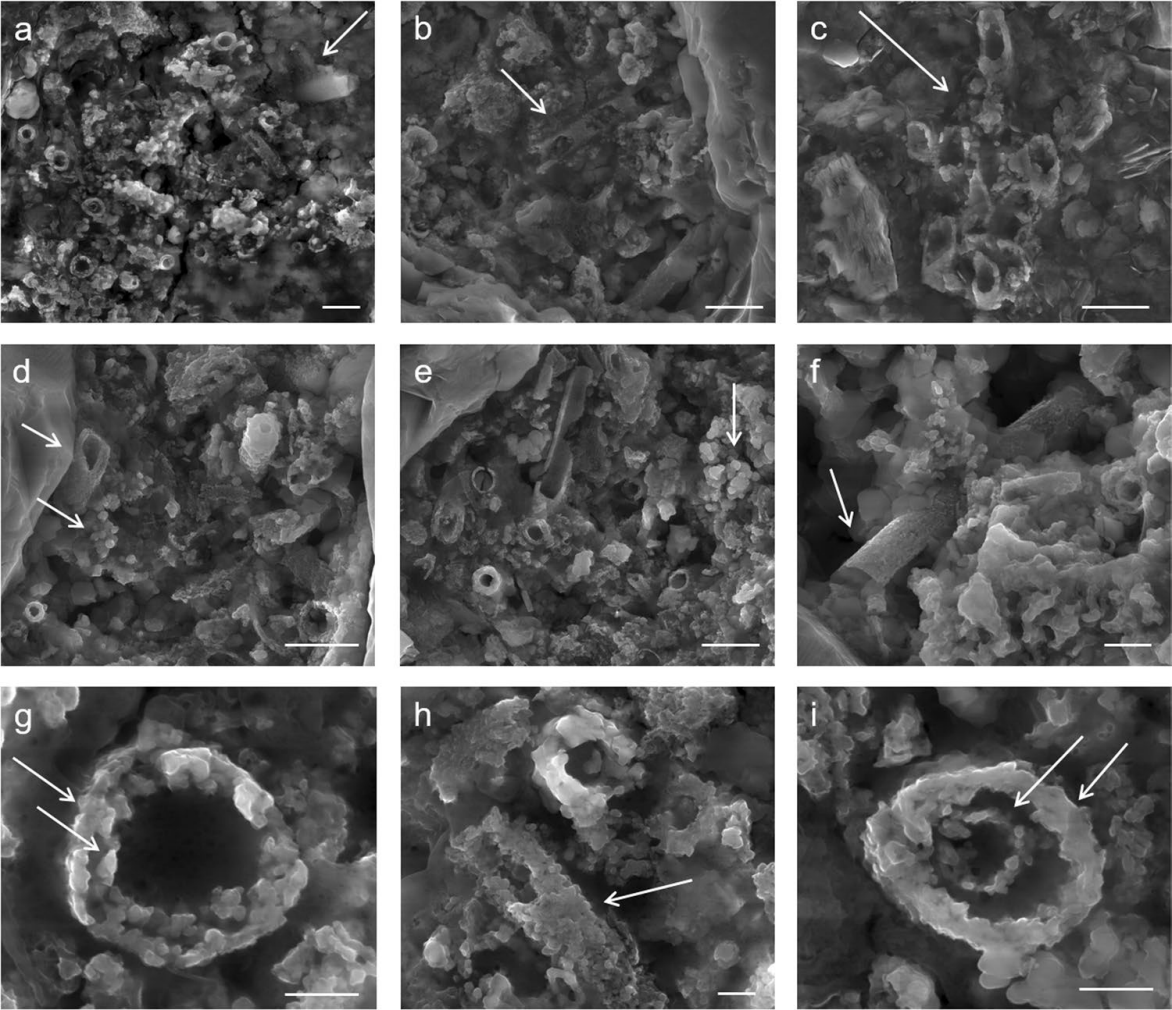
Selected micrographs from SEM examination of targeted red pigment particle in GcSi-1 rock art sample. (**a**) Concentration of broken and randomly oriented *L*. *ochracea* sheaths. Note the fractured diatom fragment in the upper right corner, and hematite and silicate microspheres distributed throughout. Scale = 2.5 µm. (**b**) Detail of *L*. *ochracea* sheath showing advanced degradation (holes) in the sidewall of a sheath body. Scale = 2.5 µm. (**c**) Detail of a small, intact cluster of *L*. *ochracea* bodies. This was the sole identifiable example of a coherent cluster in the sample. Note the warped shapes of the tube openings. Scale = 2.5 µm. (**d**,**e**) Concentrations of *L*. *ochracea* sheaths displaying fractured ends and globular, porous sheath exterior walls. Note the clusters of hematite and silicate microspheres and blocky octahedral and tetrahedral hematite polymorphs distributed through the pigment matrix. Scale = 2.5 µm. (**f**) Rare example of a relatively intact *L*. *ochracea* sheath. Scale = 1.25 µm (**g**–**i**) Details of broken sheath ends. Note the globular surface texture indicating localized melt and recrystallization of iron and silica. Specimens shown in (**g**,**i**) have double-walled inner rings, indicating maturity at the time of death. Scales = 0.5 µm.

**Figure 4:**
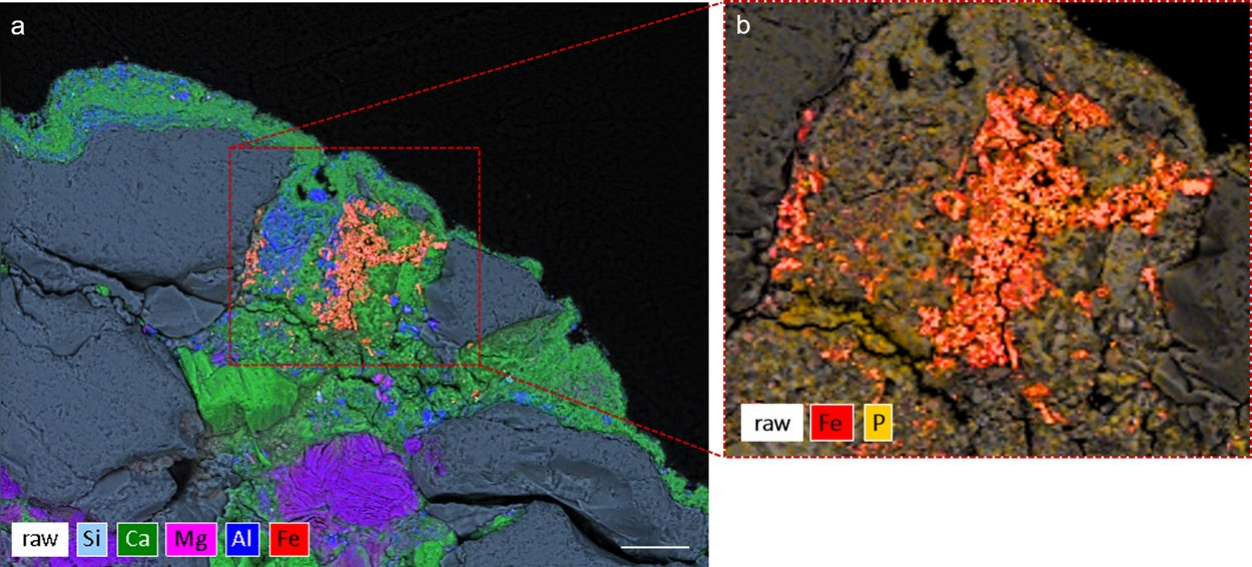
SEM-EDS Hyperspectral Map of GcSi-1. (**a)** Cross section EDS detail of a red pigment particle embedded in the calcite layer. Scale = 25 µm. (**b**) EDS map detail of pigment particle showing concentrations of iron and phosphorus.

**Figure 5:**
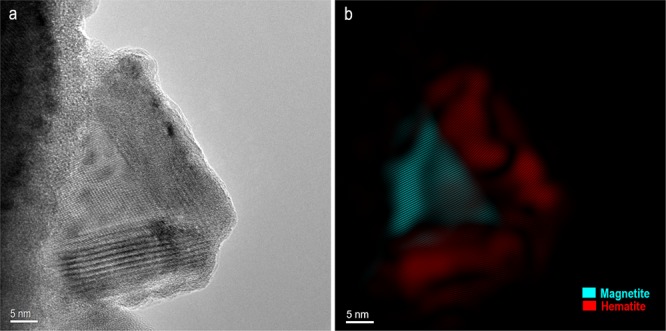
HRTEM Analysis of GcSi-1. (**a**) HRTEM image showing magnetite particle coated with hematite phase on the exterior surface of a *L*. *ochracea* sheath body. (**b**) Hematite and magnetite phases highlighted in color. Details on phase identification and image processing can be found in SI Fig. 3b. Scales = 5 nm.

The pigment used for rock paintings at Babine Lake is composed of a heterogeneous mixture of *L*. *ochracea* biomineral fossils and trace amounts of sediment impurities commonly associated with iron microbial mats (Fig. 3a–i). The biomineral sheaths exhibited clear signs of pulverization and homogenization. Sheaths were fractured and multidirectional in orientation, and showed structural and melt features (warping, slumping) characteristic of high temperature thermal exposure. External surfaces of the sheaths showed partial melting and Fe:Si phase separation, and localized crystallization of hematite and magnetite nanoparticles. SEM-EDS confirmed the bulk composition of the sheaths are a Fe:Si:P nanocomposite (Fig. 4, and see SI Fig. 2). High-resolution TEM (HRTEM) crystallography revealed the presence of magnetite, maghemite, and hematite nanoparticles that formed on the exterior and interior surfaces of the sheaths, and as localized crystallization within the sheath body matrix (Fig. 5 and see SI Fig. 3a–f). Quartz (SiO_2_) microspheres and amorphous silicates were present throughout the pigment matrix (Fig. 3d,e). Micro-Raman spectroscopy indicated the paint layer contained hematite and hydrous ferrous sulfate (FeSO_4_*H_2_O) (SI Fig. 4). Powder XRD patterns of painted and unpainted surfaces of the GcSi-1 fragment are both dominated by quartz and calcite (CaCO_3_) (attributed to silicified limestone substrate and surface accretion). The pigmented surface showed patterns for hematite and fayalite (Fe_2_SiO_4_), a high temperature iron silicate (1173 °C) known to form at temperatures as low as 890 °C in the presence of phosphorous^64^ (SI Fig. 5, SI Data 1).

At rock art sites a range of mineral phases form at the interface between rock surfaces and the atmosphere, including calcite, gypsum, silica skins, and various oxalates. Optical microscopy showed details of the depositional and diagenetic microenvironment of GcSi-1. The rock art panel was painted on an outcrop of silicified limestone covered in a calcite mineral coating ranging in thickness from 10–50 µm (see SI Fig. 6). Calcite forms as a weathering product on the surface of limestone, and the iron oxide pigment particles were stratified in the calcite coating. The paint was applied over the mineral coating and subsequently overlaid as calcite formation progressed. The red pigment layer ranged in thickness from ~5–10 µm, and where calcite accretion was notably thicker the pigment color intensity was visibly reduced. It is probable that the calcite accretion aided the long-term stabilization of the pigment by binding it to the rock substrate, effectively creating a protective skin and enhancing resistance to weathering and chemical alteration.

**Figure 6:**
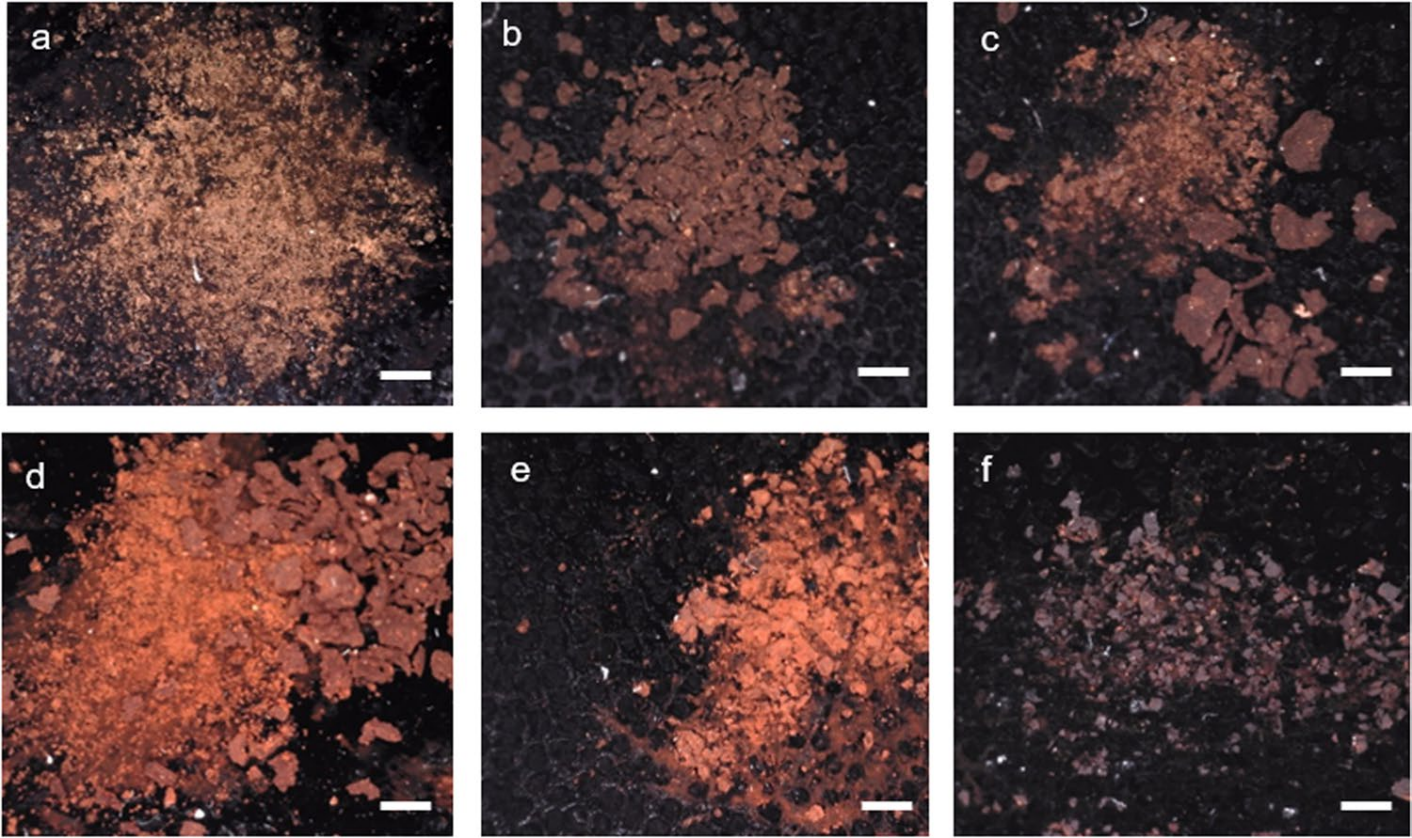
Thermally-induced Color Change of *L*. *ochracea* Control Samples. A series of digital micrographs illustrating thermally-induced color change of *L*. *ochracea.* Samples were heated in oxidizing atmosphere in a muffle furnace, held for 3 h each. Munsell scores are provided. (**a**) Untreated (2.5YR 2.5/2), (**b**) 200 °C (2.5YR 2.5/3), (**c**) 400 °C (2.5YR 3/4), (**d**) 600 °C, (2.5YR 3/6), (**e**) 800 °C (2.5YR 3/6), (**f**) 1,000 °C (10 R 2.5/1). Scale 1 = mm.

### Morphological and structural changes in *L. ochracea* FeOB controls

#### SEM examination of furnace heated samples

Heat treatment of *L*. *ochracea* control samples resulted in visible transformations in the pigment matrix color at each processing temperature from untreated through to 1,000 °C (Fig. 6). Untreated FeOB appears dull brown-orange with a matte luster and dusty texture. FeOB samples heated to 200 °C and 400 °C were similar in luster and texture, yet exhibited increasing Munsell *value* and *chroma* scores (intensification of orange-red undertones) via accelerated oxidation. Samples heated to 600 °C and 800 °C were almost identical in color, exhibited the highest intensity of vivid red-orange, and increased luster and sheen. Notably, the color of the 600 °C and 800 °C samples closest resembled that of the pigment at GcSi-1. At 1,000 °C, the pigment transitioned to purple-red and slaty-black, which we attribute to the production of FeO, Fe_3_O_4_, and remnant charred organics.

**Figure 7:**
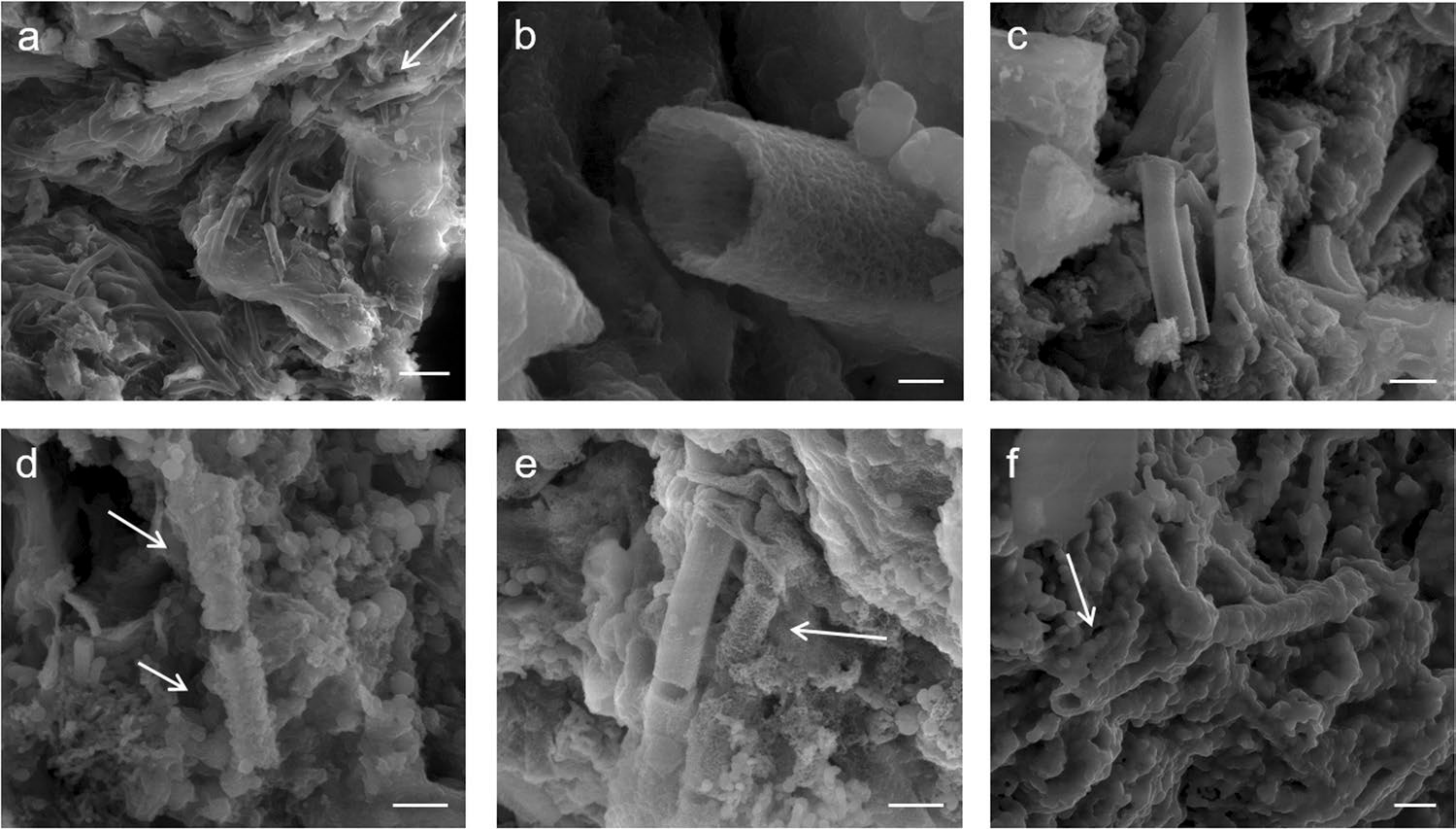
SEM Micrographs of Incrementally Heat-Treated *L*. *ochracea* Control Samples. (**a**) Untreated FeOB. Note the smooth exterior texture. Scale = 10 µm (**b**) 200 °C, note the absence of structural changes. Scale = 0.25 µm (**c**) 400 °C, note the absence of structural changes and smooth sheath exterior. Scale = 1.25 µm (**d**) 600 °C, showing structural changes including globular deposits and fraying of sheath exteriors. Scale = 0.75 µm. (**e**) 800 °C, note melt features on sheath exterior surfaces and nucleation of hematite microspheres. Scale = 1.0 µm (**f**) 1,000 °C, note the complete phase transformation to magnetite and hematite polymorphs. Scale = 1.25 µm. *An early iteration this figure was published in a non-peer-reviewed conference proceedings abstract in a special issue of the journal *Microanalysis and Microscopy*^87^.

Figure 7(a–f) highlights key morphological and structural changes in *L*. *ochracea* sheaths at increasing temperatures. The untreated sample (a) showed growth of intact *L*. *ochracea* clusters exhibiting smooth exterior textures. When heated to 200 °C (b), they remained relatively intact and showed little morphological changes. At 400 °C (c) the sheaths exhibit structural warping and fraying of exterior fibrils. At 600 °C (d), the first evidence for melt features and separation of Fe:Si bonds was observed. Note in Fig. 7d that the exterior surfaces of the sheaths begin to degrade and nucleate the formation of globular hematite and amorphous silica microspheres. At 800 °C (e), the sheaths showed continued proliferation of hematite microspheres and other iron oxide phases. We observed continued slumping, warping, and shrinkage of the entire FeOB-enriched particle mass due to hydroxide (OH) loss. At 1,000 °C (f), we observed near-complete phase transformation to crystalline hematite and magnetite polymorphs. What were once well-defined micro-filament structures were rendered hardly recognizable. Of those that were identifiable, the exterior surfaces showed complete coverage of massive, angular particles resembling tetra- and octahedral Fe-oxide polymorphs. The morphological characteristics observed in the FeOB control samples heated between 600 °C and ~800 °C closest resemble those seen in the GcSi-1 pigment.

#### SEM *in situ* heating

We performed *in situ* observation of temperature-dependent structural and morphological changes in *L*. *ochracea* biominerals using a SEM-coupled heating mantle (see SI Text 3b). The specimen was monitored and recorded over 4 hours, with holds at intermittent temperatures (500 °C, 600 °C, 700 °C, 800 °C, 900 °C, 950 °C, 1,050 °C) to capture SEM micrographs highlighting important morphological changes (see SI Data 2 and SI Video 1). Key observations were: the greatest change between ambient temperature and 500 °C was shrinkage of the pigment particle mass from structural OH loss (SI Fig. 7). Between 600 °C and 700 °C, slumping and warping of the sheath structures were observed throughout. In the range of 700 °C to 800 °C, the sheaths exhibited the appearance of “sweating” or “beading”, as Fe:Si phase separation began. Above 800 °C, the nucleation of crystalline polymorphs (hematite, magnetite, maghemite particles up to ~0.20 µm) on the exterior surfaces of the sheaths rapidly proliferated. As temperature approached 900–950 °C, degradation advanced as sheath structures continued to warp and slump. Approaching 950 °C, hematite microspheres began to coalesce into larger conglomerates up to ~1 µm in size. Between 950 °C and 1,000 °C, hematite and magnetite formation continued as the sheaths degraded, and tetra- and octahedral polymorphs formed (see SI Fig. 8). At 1,149 °C the SiN overlay of the Protochip ruptured, leaving the FeOB particle unrecoverable. A temperature hold at 1,050 °C highlights the hematite phase nucleation and separation of amorphous silica oxide (see SI Video 2). Based on the morphological transformations observed *in situ*, the control sample closest resembles the GcSi-1 pigment in the thermal range of 700 °C to 900 °C (see SI Text 3c).

### Molecular analysis of FeOB controls

Powder XRD analysis of the furnace heated FeOB control samples showed a clear trend of Fe-oxide phase transitions consistent with observations from electron microscopic methods (SI Table 1, SI Data 2, SI Text 4). Quartz was the dominant impurity in each FeOB-enriched matrix regardless of temperature. In samples heated above 600 °C, pattern matches for cristobalite were also observed. The untreated FeOB showed major and minor phases of poorly-ordered iron phosphate oxide/hydroxides. The 200 °C sample showed matches for poorly-ordered iron phosphate oxide/hydroxide, and FeO. At 400 °C, iron phases included iron phosphate, FeO, and magnetite. At 600 °C, phases included iron phosphates, magnetite, and hematite. In the 800 °C sample, the iron hydroxides and iron phosphates were lower in abundance or undetectable, and major phases included iron silicate (Fe_2_SiO_4_), hematite, and magnetite. At 1,000 °C, major phases included hematite, magnetite, and iron silicate. The key observations here are that magnetite and hematite are not readily detected in samples heated below 600 °C, that iron silicates do not form until 800 °C, and that iron hydroxides/phosphates are low or undetectable above 800 °C. By comparison, this suggests that the GcSi-1 sample must have been heated to temperatures ~800 °C to achieve a similar mineralogical profile.

### Nanoparticle phase identification by high resolution (HR-)TEM crystallography

HRTEM confirmed the structure and distribution of nanoparticles in the GcSi-1 and FeOB 800 °C control samples. A TEM lamella cross section of each specimen was prepared using a focused-ion beam scanning electron microscope (FIB-SEM) (see SI Text 2a). Examination of individual particles in both specimens showed that the FeOB sheaths had a mixture of magnetite, maghemite, and hematite nanoparticles on their interior and exterior surfaces. Figure 5 (and see SI Fig. 3a) shows a magnetite nanoparticle precipitated on the exterior surface of the FeOB sheath, which itself is coated in a layer of crystalline hematite. Other regions of interest in both specimens exhibited similar magnetite and hematite nanoparticles and globules of amorphous silicate distributed throughout the pigment matrix, suggesting that the FeOB control and GcSi-1 were heated to a comparable temperature range of ~800 °C (see SI Fig. 3a–h).

### Magnetic properties: SQUID magnetometry

Iron-oxide mineral compounds change their magnetic properties with temperature exposure. Natively, *L*. *ochracea* sheaths do not exhibit magnetic properties^65^; however, upon heating to temperatures sufficient to induce phase conversion they can exhibit characteristic magnetic properties^66^. The process of thermally-induced magnetization in FeOB-enriched sediment is complex and not fully understood. Examples of abiotic Fe-oxide magnetization^67–70^, and soil iron minerals^71^ are common, though none demonstrate comparable proxies for interpreting magnetic properties of *L*. *ochracea*.

**Figure 8:**
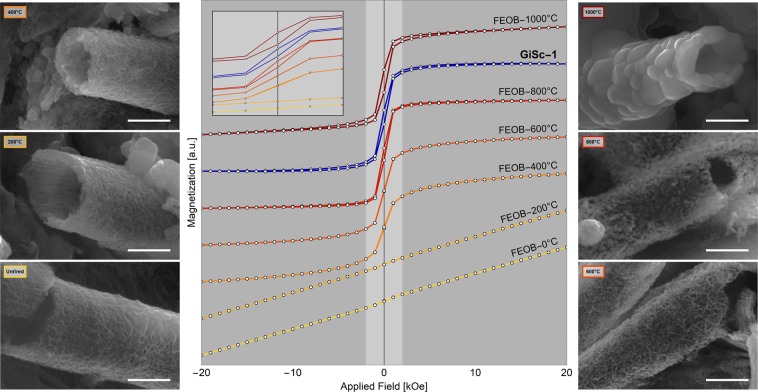
SQUID magnetometry and associated *L. ochracea* sheath transformation. Hysteresis loops for GcSi-1 and *L*. *ochracea* control samples (center). SEM micrographs (left and right) showing the morphological transitions of *L*. *ochracea* sheaths corresponding each thermal gradient. Scale = 1.0 µm.

To develop a model for thermally-induced magnetization of biogenic Fe-oxide, we tested the GcSi-1 and heat-treated *L*. *ochracea* control samples using SQUID magnetometry. We hypothesized that if the biomineral pigment used at GcSi-1 had been fired above at least 600 °C, this would be observable in its magnetic properties. Figure 8 shows hysteresis loops illustrating the magnetic response for each FeOB control sample and GcSi-1 (see also SI Data 3). The untreated and 200 °C FeOB samples were paramagnetic. The 400 °C and 600 °C samples both showed magnetic saturation at less than 3 kOe applied field, with no hysteresis, consistent with the presence of some superparamagnetic Fe_2_O_3_ nanoparticles. This conforms to our SEM observation of Fe:Si phase separation and recrystallization initiating at ~600 °C. Recall that from observations with SEM the iron oxide particles began to agglomerate into larger particles (~1 µm) at 800 °C. This is evidenced by a sharper saturation curve and by the appearance of hysteresis in the measurement of the 800 °C sample as the particles became large enough to display weak ferromagnetism. At 1,000 °C the saturation magnetization of the FeOB sample drops by an order of magnitude. The GcSi-1 pigment sample does not exhibit characteristics of strictly paramagnetic materials, indicating that the FeOB was minimally heated above 200 °C. The saturation curve is sharp, akin to the 600 °C and 800 °C control samples, and hysteresis is most similar to that seen in the 800 °C control sample.

SQUID magnetometry results reveal insights for modelling magnetic responses to thermal alteration. A pigment sample composed of untreated *L*. *ochracea* would display characteristics of paramagnetism, whereas a sample matrix existing of entirely magnetite would exhibit characteristics of superparamagnetism. An impure sample of mixed phases can produce complex results. The GcSi-1 pigment showed characteristics of both that would be consistent with a mixture containing paramagnetic hematite and nanoparticle-sized “hot spots” of superparamagnetic magnetite. This is consistent with our observations that the paint is a mixture of materials with incomplete phase transformations, indicating that the pigment would have reached the minimum temperature for magnetite formation, yet would not have exceeded temperatures between ~800 °C and 1,000 °C. If so, the GcSi-1 sample would have not only exhibited stronger characteristics of superparamagnetism, but the paint would have also been charred to a grey-black hue. The magnetometry tests strongly suggest a likely temperature range of at least above 600 °C, and no greater than just above 800 °C.

## Discussion

### Reconstructing thermal history of FeOB biomineral pigments used at Babine Lake

In summary, our control study has provided strong evidence that the biomineral pigment used at GcSi-1 was likely heated to a range of 750 °C to 850 °C as part of the paint preparation *chaîne opératoire*. The characteristics that we observed in both furnace and SEM *in situ* heating regimes suggested that phase separation and hematite and magnetite nucleation in *L*. *ochracea* biomineral sheaths occurred as low as 600 °C in oxidizing conditions. Slumping, warping, and degradation of sheaths begins at ~400 °C and intensifies through to >1,000 °C. Importantly, at 800 °C, hematite and magnetite nanoparticles rapidly form on the surfaces of the *L*. *ochracea* sheaths. Our study of the GcSi-1 sample by micro-Raman and XRD showed fayalite and hematite, but not magnetite or maghemite. However, particle-specific HRTEM enabled the identification of hematite, magnetite, and maghemite nanoparticles. We attribute this to differences in interaction volume between XRD (mm to micron scale) and HRTEM (nanoscale), as most magnetite, maghemite, and hematite particles were nano-sized. The heterogeneous mixture of iron oxide phases is consistent with trends observed by SQUID magnetometry, where results suggested the presence of multiple mineral phases of differing Fe + valencies and stoichiometries, each with specific magnetic properties. The overall changes in magnetic properties seen in the FeOB control samples, and GcSi-1 by comparison, are consistent with the precipitation and growth in size of hematite, magnetite, and maghemite nanoparticles.

### Could the FeOB proliferate *in situ* at GcSi-1?

Upon our initial identification of *L*. *ochracea* we considered the possibility that the bacteria colony could have grown on the surface of the rock art panel. This is highly unlikely, if not impossible. If the *L*. *ochracea* grew *in situ* at the open-air GcSi-1 pictograph, their morphological characteristics would appear differently than they do. Natively, *L*. *ochracea* grow under water in highly ordered microbial mats^31^. In the pigment from GcSi-1, the *L*. *ochracea* biominerals are disaggregated, fractured, and randomly oriented in such a way that could only be achieved by deliberate pulverization, as typical during the processing of mineral paints. Moreover, the exposed, open-air rock wall face is a microenvironment that is not habitable to *L*. *ochracea* or conducive to its proliferation. These bacteria require a continuous aqueous source of ferrous iron to grow^72^, yet the underlying mineral strata, silicified limestone, is not iron-rich. If they were natural growths one would expect to see evidence for *L*. *ochracea* on other areas of the rock wall adjacent to the painting. Yet, in our screening we did not observe evidence for *L*. *ochracea* biominerals in any location other than intermixed in the red pigment particles. Moreover, the presence of shattered freshwater diatoms mixed into the pigment matrix further supports that the *L*. *ochracea* mass did not develop *in situ*, but most likely derived from a shallow surficial water source, typical of where *L*. *ochracea* grows naturally^73^.

### Implications for pyrotechnology and resource use at Babine Lake

A growing body of evidence indicates that Pacific Northwest peoples were active stewards of local landscapes and resources, including plant management systems^74–77^, anthropogenic burning and forest management^32^, and aquatic resource management^78^. Those practices were developed over millennia in adaptive response to seasonal and annual fluctuations in resource availability typical of the Canadian Plateau northern temperate region. The regular use of fire is an obvious essential component of daily life including residential heating, food preservation, cooking, ceremonial activities^79^, and, as demonstrated here, thermal enhancement of raw materials used to produce paints. Two main types of hearths were commonplace: closed earth ovens and open cooking fires. Closed earth ovens, sometimes referred to as steaming pits, were subterranean pits used to steam or slow-cook large quantities of food at low temperatures over long periods. A large fire would be constructed in a shallow pit lined with cobble-sized rocks used to retain heat for several days. Foods would be wrapped in skunk cabbage leaves, tree bark, or fern fronds, and covered in earth for insulation^80^. The maximum temperatures achieved by pit fires were deliberately low (<100 °C)^77^, therefore not sufficient to thermally enhance *L*. *ochracea* pigment. Open, domestic cooking hearths were larger and typically contained mixed soft and hardwood fuel and cobble-sized rocks for heat retention^81^. In addition to food preparation, they were used for domestic heating and would have been continuously fueled, enabling higher temperatures to be reached^82^. In an extensive metadata survey, Wolf *et al*.^83^ found that average temperatures of naturally occurring fires in grassland (283 °C ± 134 °C), shrubland (503 °C ± 211 °C), and mixed forest (287 °C ± 151 °C), were lower, while domestic “woody fuel” fires (797 °C ± 165 °C) usually exceeded 630 °C. This suggests that open domestic hearth fires were a suitable environment for thermal enhancement of FeOB pigment, and precludes the possibility that a forest fire would have been responsible for the thermal alteration characteristics observed in GcSi-1 paint.

Our results show that the paint-makers at Babine Lake harvested iron-enriched aquatic microbial mats dominated by *L*. *ochracea*, which were skillfully heat treated as part of the pigment production *chaîne opératoire*. Small masses of the *L*. *ochracea*-enriched sediment were treated by controlled, indirect heating before being ground to powder. The rate of heating would have varied depending on proximity to the heat source, and the size, shape, and density of the sediment grains. Variable particle sizes and their gradient surface exposure to heat would have resulted in the heterogeneous phase transformation that we observed in our control samples and GcSi-1. The individuals responsible for preparing the paints knew how controlled temperatures ranging from 750 °C to 850 °C would enhance the sediment mass color to achieve a vibrant red, and improve colorfastness, stability, and resistance to degradation. They also knew to avoid rapid heating and cooling, or charring at temperatures exceeding ~900 °C. Our analyses of the pigments used at Babine Lake indicate that late Holocene hunter-gatherers possessed skilled knowledge of FeOB properties, and were selective in their use of durable and highly thermostable biomineral pigments.

## Methods

### GcSi-1 (Boling Point) rock art

The GcSi-1 fragment was collected during archaeological survey by Mohs and Mohs^84^ and archived at Royal British Columbia Museum (Victoria, Canada). A small fragment (~4 mm × 1 mm) of the pigmented portion was carefully removed, embedded in epoxy resin, cut in cross section, and polished with 15 µm diamond grit.

### FeOB control samples

Samples of aqueous microbial iron mats (approximately 40 ml each) dominated by the species *L*. *ochracea* were collected from a freshwater stream in Carrabasset Valley, Maine (45.08; -70.28), see SI Text 2. Samples were evaporated at ambient temperature in three sterile 50 ml Falcon tubes, resulting in orange-brown, coarse-grained sediment. The sample mass was divided in preparation for muffle furnace and SEM heating procedures.

### Heating procedure: muffle furnace

A portion of the untreated *L*. *ochracea* sediment mass was further divided into six sub-samples (approx. 5 ml each) in porcelain crucibles. One sub-sample was reserved as untreated. The remaining five were each individually held for 3 hours under oxidizing conditions in a muffle furnace to maximum temperatures of 200 °C, 400 °C, 600 °C, 800 °C, and 1,000 °C, respectively, and allowed to cool to ambient temperature. The six control samples were subsequently examined by SEM, XRD, and SQUID magnetometry.

### Heating procedure: *in situ* SEM

This consisted of a continuous *in situ* heating using Protochips™ (Aduro 350), a SEM-coupled heating platform designed to precisely control temperature up to 1200 °C. The MEMS-based heating chip contained a SiN overlay directly atop the heated region. The procedure took place over 4 h 8 m. Temperature was ramped at a rate of 0.5 °C per second using closed-loop temperature control, with intermittent holds at 500 °C, 600 °C, 700 °C, 800 °C, 900 °C, 950 °C, and 1,050 °C to capture SEM images (see SI Text 1b, SI Data 3, SI Videos 1–3).

### Powder XRD

FeOB control samples were analyzed as homogenized powders. The GcSi-1 fragment was analyzed intact, targeting both pigmented and non-pigmented sides. XRD patterns were collected using a Scintag X2 powder diffractometer equipped with a Peltier-cooled energy sensitive detector operating at 40 kV and 50 mA, using Cu-Kα radiation (1.54060 Å). A monochromatic X-ray beam was oriented at each target sample, and scanned from 5° to 80° 2ϴ at a scanning step size of 0.02°, and a dwell time of 2.0 s each. The peak patterns were identified with the aid of FullProf and Match Phase Identification software (version 3.7.1.132), and compared to Crystallography Open Database^85^ and RRUFF Database^86^.

### Electron microscopy methods

Initial examination of GcSi-1 was performed on a FEI Quanta 600 F environmental scanning electron microscope (ESEM) equipped with a Bruker Quantax 200 silicon drift detector for X-ray energy dispersive spectrometry. The ESEM was operated in low vacuum mode and hyperspectral EDS data were collected at 15 kV. This procedure was used to examine mineral accretionary deposits, paint thickness and composition, and to produce elemental maps. Cross-sectional lamella preparation was performed on a FEI Scios Analytical DualBeam FIB-SEM and EDS spectra (SI Fig. 2) were collected using an Oxford X-Max 50 SDD. A focused Ga^2+^ ion beam and nanomanipulator were used to prepare a ~60 nm thin section of the GcSi-1 sample for transmission electron microscopy (TEM) (see SI Video 3, SI Fig. 10). The same procedure was used to prepare a thin section of the FeOB control sample heated to 800 °C. Both TEM lamellas were subsequently examined using a FEI Tecnai F30 Twin to perform high resolution TEM imaging at 300 kV.

### Micro-Raman spectroscopy

Micro-Raman spectroscopy was performed at University of Missouri using a Renishaw inVia Raman microscope equipped with a 633 nm laser source. We identified target areas at low magnification (10x objective lens), while spectral analysis was done using a 50x objective lens. Spectral data were collected at a spot size of 1 µm, targeting a spectral range of 100–1300 cm^−1^ (wavelength). Multiple measurements were taken at low laser output (0.1–5%) for 10 and 20 seconds to minimize sample damage. Spectral patterns were matched using reference spectra available through RRUFF database. In addition to Raman spectroscopy we attempted ATR-FTIR on all samples with inconclusive results (see SI Text 6).

### SQUID magnetometry

Magnetic hysteresis measurements were performed using a Quantum Design Magnetic Properties Measurement System (MPMS) at the Brockhouse Institute for Materials Research, McMaster University. Powdered FeOB samples were loaded into gelatin capsules inside a plastic straw with empty capsules above and below the sample to reduce the background contribution from gelatin. Magnetization at 300 K was measured as a function of applied field in the range of +/− 20 kOe and normalized to the sample mass. The GcSi-1 sample was mounted on a quartz rod for measurement. To remove the diamagnetic contribution of the rock substrate, the slope of the high field sections of the hysteresis plot was subtracted from the data set to show the magnetic contribution of the pigment. No mass normalization was performed as the mass of pigment could not be determined without destroying the sample (see SI Text 4, SI Data 3).

## Supplementary information

Supplementary Text

SI Video 1

SI Video 3

SI Video 2

SI Data 1

SI Data 2

SI Data 3

## Data Availability

The authors declare that all data generated and analyzed during this study are included in this published article and its supplementary information files.

## Code availability

The authors declare that no custom code or mathematical algorithms were used in this study.

## References

[1] Brooks AS (2018). Long-distance stone transport and pigment use in the earliest Middle Stone Age. Science.

[2] Marean CW (2007). Early human use of marine resources and pigment in South Africa during the Middle Pleistocene. Nature.

[3] Henshilwood CS (2011). A 100,000-Year-Old Ochre-Processing Workshop at Blombos Cave, South Africa. Science.

[4] Roebroeks W (2012). Use of red ochre by early Neandertals. Proceedings of the National Academy of Sciences.

[5] Hoffmann DL, Angelucci DE, Villaverde V, Zapata J, Zilhão J (2018). Symbolic use of marine shells and mineral pigments by Iberian Neandertals 115,000 years ago. Science Advances.

[6] Sajó IE (2015). Core-Shell processing of natural pigment: upper Palaeolithic red ochre from Lovas, Hungary. PloS one.

[7] Zilhão J (2010). Symbolic use of marine shells and mineral pigments by Iberian Neandertals. Proceedings of the National Academy of Sciences.

[8] Aubert M (2018). Palaeolithic cave art in Borneo. Nature.

[9] Brumm A (2017). Early human symbolic behavior in the Late Pleistocene of Wallacea. Proceedings of the National Academy of Sciences.

[10] Erlandson JM (2011). Paleoindian seafaring, maritime technologies, and coastal foraging on California’s Channel Islands. Science.

[11] Lahren L, Bonnichsen R (1974). Bone foreshafts from a Clovis burial in southwestern Montana. Science.

[12] Stafford MD, Frison GC, Stanford D, Zeimans G (2003). Digging for the color of life: Paleoindian red ochre mining at the Powars II site, Platte County, Wyoming, USA. Geoarchaeology.

[13] Smallwood AM, Jennings TA, Pevny CD (2018). Expressions of ritual in the Paleoindian record of the Eastern Woodlands: Exploring the uniqueness of the Dalton cemetery at Sloan, Arkansas. Journal of Anthropological Archaeology.

[14] Lemke AK, Wernecke DC, Collins MB (2015). Early art in North America: Clovis and Later Paleoindian incised artifacts from the Gault site, Texas (41BL323). American Antiquity.

[15] Speth JD, Newlander K, White AA, Lemke AK, Anderson LE (2013). Early Paleoindian big-game hunting in North America: provisioning or politics?. Quaternary International.

[16] Potter BA, Irish JD, Reuther JD, Gelvin-Reymiller C, Holliday VT (2011). A terminal Pleistocene child cremation and residential structure from eastern Beringia. Science.

[17] Arnett C, Morin J (2018). The Rock Painting/Xela: ls of the Tsleil-Waututh: A Historicized Coast Salish Practice. Ethnohistory.

[18] Bishop, C. A. Coast-interior exchange: the origins of stratification in Northwestern North America. *Arctic Anthropology*, 72–83 (1987).

[19] Carlson, R. L. In *Time and space: dating and spatial considerations in rock art research* 7–12 (1993).

[20] Erlandson JM, Robertson JD, Descantes C (1999). Geochemical analysis of eight red ochres from western North America. American Antiquity.

[21] MacDonald BL (2013). Elemental analysis of ochre outcrops in southern British Columbia, Canada. Archaeometry.

[22] MacDonald BL, Hancock RGV, Cannon A, Pidruczny A (2011). Geochemical characterization of ochre from central coastal British Columbia, Canada. Journal of Archaeological Science.

[23] Corner, J. *Pictographs* (*Indian rock paintings*) *in the Interior of British Columbia*. (Wayside Press, 1968).

[24] Wainwright IN (1985). Rock art conservation research in Canada. Bollettino del Centro Camuno di studi preistorici.

[25] York, A., Daly, R. & Arnett, C. *They write their dreams on the rock forever: rock writings of the Stein River Valley of British Columbia*. (Talonbooks, 1993).

[26] Arnett, C. A. *Rock art of Nlaka’pamux: indigenous theory and practice on the British Columbia Plateau*, University of British Columbia (2016).

[27] Morice, A. G. *The History of the Northern Interior of British Columbia 1660-1880*. (Ye Gallon Press, 1906).

[28] Ames, K. M. Report of Excavations at GhSv 2, Hagwilget Canyon. *GF MacDonald and RI Inglis*, *Mercury Series*, *Archaeological Survey Papers* **87** (1979).

[29] Reimer R (2015). Reassessing the role of Mount Edziza obsidian in northwestern North America. Journal of Archaeological Science: Reports.

[30] Mitchell D, Donald L (1988). Archaeology and the study of Northwest Coast economies. Prehistoric Economies of the Pacific Northwest Coast, Research in Economic Anthropology.

[31] Chan CS (2016). The architecture of iron microbial mats reflects the adaptation of chemolithotrophic iron oxidation in freshwater and marine environments. Frontiers in microbiology.

[32] Hoffmann T (2016). Engineered feature used to enhance gardening at a 3800-year-old site on the Pacific Northwest Coast. Science advances.

[33] Hashimoto H (2012). Preparation, microstructure, and color tone of microtubule material composed of hematite/amorphous-silicate nanocomposite from iron oxide of bacterial origin. Dyes and Pigments.

[34] Fiuza TER (2018). Iron-based inorganic pigments from residue: Preparation and application in ceramic, polymer, and paint. Dyes and Pigments.

[35] Chalmin, E. & Huntley, J. (Oxford Handbook of Archaeology, 2017).

[36] Dayet L, Le Bourdonnec F-X, Daniel F, Porraz G, Texier P-J (2016). Ochre provenance and procurement strategies during the Middle Stone Age at Diepkloof Rock Shelter, South Africa. Archaeometry.

[37] Huntley J, George S, Sutton M-J, Taҫon P (2018). Second-hand? Insights into the age and ‘authenticity’of colonial period rock art on the Sunshine Coast, Queensland, Australia. Journal of Archaeological Science: Reports.

[38] Velliky EC, Porr M, Conard NJ (2018). Ochre and pigment use at Hohle Fels cave: Results of the first systematic review of ochre and ochre-related artefacts from the Upper Palaeolithic in Germany. PloS one.

[39] Marcaida I (2019). Raman microscopy as a tool to discriminate mineral phases of volcanic origin and contaminations on red and yellow ochre raw pigments from P ompeii. Journal of Raman Spectroscopy.

[40] d’Errico F, Moreno RG, Rifkin RF (2012). Technological, elemental and colorimetric analysis of an engraved ochre fragment from the Middle Stone Age levels of Klasies River Cave 1, South Africa. Journal of Archaeological Science.

[41] Henshilwood CS (2018). An abstract drawing from the 73,000-year-old levels at Blombos Cave, South Africa. Nature.

[42] Hodgskiss T, Wadley L (2017). How people used ochre at Rose Cottage Cave, South Africa: Sixty thousand years of evidence from the Middle Stone Age. PloS one.

[43] Wadley L, Hodgskiss T, Grant M (2009). Implications for complex cognition from the hafting of tools with compound adhesives in the Middle Stone Age, South Africa. Proceedings of the National Academy of Sciences.

[44] Prinsloo LC, Tournié A, Colomban P, Paris C, Bassett ST (2013). In search of the optimum Raman/IR signatures of potential ingredients used in San/Bushman rock art paint. Journal of Archaeological Science.

[45] Villa P (2015). A milk and ochre paint mixture used 49,000 years ago at Sibudu, South Africa. PloS one.

[46] Roldán C, Murcia-Mascarós S, López-Montalvo E, Vilanova C, Porcar M (2018). Proteomic and metagenomic insights into prehistoric Spanish Levantine Rock Art. Scientific reports.

[47] Cavallo G (2018). Heat Treatment of Mineral Pigment During the Upper Palaeolithic in North‐East Italy. Archaeometry.

[48] Salomon H, Vignaud C, Lahlil S, Menguy N (2015). Solutrean and Magdalenian ferruginous rocks heat-treatment: accidental and/or deliberate action?. Journal of Archaeological Science.

[49] Hunt A (2016). The characterisation of pigments used in X-ray rock art at Dalakngalarr 1, central-western Arnhem Land. Microchemical Journal.

[50] Emerson D, Fleming EJ, McBeth JM (2010). Iron-oxidizing bacteria: an environmental and genomic perspective. Annual review of microbiology.

[51] Hashimoto H (2007). Characteristics of hollow microtubes consisting of amorphous iron oxide nanoparticles produced by iron oxidizing bacteria, Leptothrix ochracea. Journal of Magnetism and Magnetic Materials.

[52] Banfield JF, Welch SA, Zhang H, Ebert TT, Penn RL (2000). Aggregation-based crystal growth and microstructure development in natural iron oxyhydroxide biomineralization products. Science.

[53] Vesenka J, Havu J, Hruby K, Emerson D (2018). A model for sheath formation coupled to motility in Leptothrix ochracea. Geomicrobiology journal.

[54] Fortin D, Langley S (2005). Formation and occurrence of biogenic iron-rich minerals. Earth-Science Reviews.

[55] Posth NR (2013). Simulating Precambrian banded iron formation diagenesis. Chemical Geology.

[56] Krepski S, Emerson D, Hredzak‐Showalter P, Luther Iii G, Chan C (2013). Morphology of biogenic iron oxides records microbial physiology and environmental conditions: toward interpreting iron microfossils. Geobiology.

[57] Pomiès MP, Menu M, Vignaud C (1999). Red palaeolithic pigments: natural hematite or heated goethite?. Archaeometry.

[58] Rzepa G, Bajda T, Gaweł A, Debiec K, Drewniak L (2016). Mineral transformations and textural evolution during roasting of bog iron ores. Journal of Thermal Analysis and Calorimetry.

[59] Walter D, Buxbaum G, Laqua W (2001). The mechanism of the thermal transformation from goethite to hematite. Journal of Thermal Analysis and Calorimetry.

[60] Capel J, Huertas F, Pozzuoli A, Linares J (2006). Red ochre decorations in Spanish Neolithic ceramics: A mineralogical and technological study. Journal of Archaeological Science.

[61] Godfrey-Smith DI, Ilani S (2004). Past thermal history of goethite and hematite fragments from Qafzeh Cave deduced from thermal activation characteristics of the 110 C TL peak of enclosed quartz grains. ArchéoSciences, revue d’Archéométrie.

[62] Mastrotheodoros G, Beltsios K, Zacharias N (2010). Assessment of the production of antiquity pigments through experimental treatment of ochres and other iron based precursors. Mediterranean Archaeology and Archaeometry.

[63] Cudennec Y, Lecerf A (2006). The transformation of ferrihydrite into goethite or hematite, revisited. Journal of Solid State Chemistry.

[64] Yuan Q, Xu G, Zhou M, He B, Hu H (2017). The effect of p on the microstructure and melting temperature of Fe2SiO4 in silicon-containing steels investigated by *in situ* observation. Metals.

[65] Safarik I, Angelova R, Baldikova E, Pospiskova K, Safarikova M (2017). Leptothrix sp. sheaths modified with iron oxide particles: Magnetically responsive, high aspect ratio functional material. Materials Science and Engineering: C.

[66] Sayed FN, Polshettiwar V (2015). Facile and sustainable synthesis of shaped iron oxide nanoparticles: effect of iron precursor salts on the shapes of iron oxides. Scientific reports.

[67] Michel FM (2010). Ordered ferrimagnetic form of ferrihydrite reveals links among structure, composition, and magnetism. Proceedings of the National Academy of Sciences.

[68] Mooney SD, Geiss C, Smith MA (2003). The use of mineral magnetic parameters to characterize archaeological ochres. Journal of Archaeological Science.

[69] Tsatskin A, Gendler TS (2016). Identification of “red ochre” in soil at Kfar HaHoresh Neolithic site, Israel: Magnetic measurements coupled with materials characterization. Journal of Archaeological Science: Reports.

[70] Zergenyi R, Hirt A, Zimmermann S, Dobson J, Lowrie W (2000). Low‐temperature magnetic behavior of ferrihydrite. Journal of Geophysical Research: Solid Earth.

[71] Hanesch M (2009). Raman spectroscopy of iron oxides and (oxy) hydroxides at low laser power and possible applications in environmental magnetic studies. Geophysical Journal International.

[72] Fleming E (2018). Insights into the fundamental physiology of the uncultured Fe-oxidizing bacterium Leptothrix ochracea. Appl. Environ. Microbiol..

[73] Van Veen W, Mulder E, Deinema MH (1978). The Sphaerotilus-Leptothrix group of bacteria. Microbiological Reviews.

[74] Lepofsky D, Lertzman K (2008). Documenting ancient plant management in the northwest of North America. Botany.

[75] Lyons N, Ritchie M (2017). The Archaeology of Camas Production and Exchange on the Northwest Coast: With Evidence from a Sts’ ailes (Chehalis) Village on the Harrison River, British Columbia. Journal of Ethnobiology.

[76] Gottesfeld LMJ (1994). Aboriginal burning for vegetation management in northwest British Columbia. Human Ecology.

[77] Peacock SL (2008). From complex to simple: balsamroot, inulin, and the chemistry of traditional Interior Salish pit-cooking technology. Botany.

[78] Hoffman, K. M. *13*,*000 years of fire activity in a temperate rainforest on the Central Coast of British Columbia*, *Canada* PhD thesis, University of Victoria, (2018).

[79] Graesch AP, DiMare T, Schachner G, Schaepe DM, Dallen J (2014). Thermally modified rock: The experimental study of “fire-cracked” byproducts of hot rock cooking. North American Archaeologist.

[80] K’San, t. P. o. *Gathering what the Great Nature Provided: Food Traditions of the Gitksan*. (Douglas and McIntyre, 1980).

[81] Ormerod, P. L. *Reading the earth: multivariate analysis of feature functions at Xa: ytem* (*The Hatzic Rock Site*, *DgRn 23*), *British Columbia*, University of British Columbia, (2002).

[82] Werts S, Jahren A (2007). Estimation of temperatures beneath archaeological campfires using carbon stable isotope composition of soil organic matter. Journal of Archaeological Science.

[83] Wolf M (2013). Towards reconstruction of past fire regimes from geochemical analysis of charcoal. Organic Geochemistry.

[84] Mohs, A. & Mohs, G. Babine Lake Archaeological Survey Project, 1976-5. (British Columbia Heritage Conservation Branch, Victoria, British Columbia, Canada, 1976).

[85] Gražulis S (2009). Crystallography Open Database–an open-access collection of crystal structures. Journal of Applied Crystallography.

[86] Lafuente, B., Downs, R., Yang, H. & Stone, N. The power of databases: the RRUFF project. In “Highlights in mineralogical crystallography”, Armbruster, T. & Danisi, RM, eds. W. *De Gruyter*, *Berlin*, *Germany* **1**, 30 (2015).

[87] MacDonald BL (2018). Reconstructing Rock Art Fe-oxide Pigment Pyrotechnology Using *In Situ* SEM Heating Experiments. Microscopy and Microanalysis.

