# Supplementary material for: Hunter-Gatherers Harvested and Heated Microbial Biogenic Iron Oxides to Produce Rock Art Pigment
Source: Sci Rep. 2019 Nov 19;9:17070. doi: 10.1038/s41598-019-53564-w (PMC6864057; doi:10.1038/s41598-019-53564-w)
Supplement: Supplementary file 1 — Supplementary Text [file 41598_2019_53564_MOESM1_ESM.docx]

**Supplementary Materials for:**

**Hunter-Gatherers Harvested and Heated Microbial**

**Biogenic Iron Oxides to Produce Rock Art Pigment**

**Brandi Lee MacDonald,^1^* David Stalla,^2^ Xiaoqing He,^2&6^ Farid Rahemtulla,^3^ David Emerson,^4^ Paul A. Dube,^5^ Matthew R. Maschmann,^6^ Catherine E. Klesner,^7^ Tommi A. White^2&8^**

^1^Archaeometry Laboratory, University of Missouri Research Reactor, Columbia, MO 65211, USA.

^2^Electron Microscopy Core, University of Missouri, Columbia, MO 65211, USA.

^3^Department of Anthropology, University of Northern British Columbia, Prince George, BC, V2N4Z9, Canada.

^4^Bigelow Laboratory for Ocean Sciences, East Boothbay, ME 04544, USA.

^5^Brockhouse Institute for Materials Research, McMaster University, Hamilton, L8S4M1, Canada.

^6^Mechanical and Aerospace Engineering, University of Missouri, Columbia, MO 65211, USA.

^7^Department of Materials Science and Engineering, University of Arizona, Tucson, AZ 87521, USA.

^8^Biochemistry, University of Missouri, Columbia, MO 65211, USA.

*Corresponding author: [MacDonaldB@missouri.edu](mailto:MacDonaldB@missouri.edu)

**Readers are encouraged to view high-resolution versions of all figures and videos uploaded to the Figshare repository. Please follow the links where indicated.**

**This PDF file includes:**

Supplementary Text - S1 to S6

Supplementary Figures - S1 to S12 (https://figshare.com/s/84185bb749a4213a6382)

Supplementary Table 1

Supplementary Information References

**Other Supporting Online Material Available via Figshare:**

Supplementary Data 1: https://figshare.com/s/c1fa8288391a212c6c5a

Supplementary Data 2: https://figshare.com/s/0e975ff61db99cba8367

Supplementary Data 3: https://figshare.com/s/006fb5880e4e9c92c833

Supplementary Video 1: https://figshare.com/s/e6337b7466ba779f826b

Supplementary Video 2: https://figshare.com/s/28df08be5029d311e4bb

Supplementary Video 3: https://figshare.com/s/fde9b70eabc4f9d729e8

**List of Contents**

**SI Text**

SI Text 1: Rock Art and Archaeology at Babine Lake, British Columbia, Canada

SI Text 2: FeOB habitat and biogeochemistry; sampling protocol for *L. ochracea* controls

SI Text 3: Notes on SEM *in situ* videos

3a: FIB-SEM Sample Preparation

3b: Observational Notes on *in situ* heating procedure

3c: Comparison of Furnace Heating vs. Protochip Heating

SI Text 4: Expanded description of FeOB XRD data

SI Text 5: SQUID Magnetometry

SI Text 6: ATR-FTIR Analysis

**SI Figures and Table**

SI Figure 1: Selected images of Babine Lake rock art.

SI Figure 2: SEM-EDS hyperspectral map of GcSi-1.

SI Figure 3a: TEM image of a cross-sectional lamella from GcSi-1 sample, prepared for HRTEM analysis.

SI Figure 3b: Localized hematite and magnetite nanoparticle precipitated on the surface of a *L. ochracea* sheath in the GcSi-1 sample.

SI Figure 3c: Localized hematite nanoparticle precipitated on the surface of a *L. ochracea* sheath in the GcSi-1 sample.

SI Figure 3d: Localized magnetite nanoparticle precipitated on the surface of a *L. ochracea* sheath in the GcSi-1 sample.

SI Figure 3e: Localized maghemite nanoparticle precipitated on the surface of a *L. ochracea* sheath in the GcSi-1 sample.

SI Figure 3f: Localized maghemite nanoparticle precipitated on the surface of a *L. ochracea* sheath in the GcSi-1 sample.

SI Figure 3g: Cross-section of *L. ochracea* sheath from FeOB 800°C control sample, prepared for HRTEM analysis.

SI Figure 3h: Localized magnetite nanoparticle precipitated on the surface of a *L. ochracea* sheath in the FeOB 800°C control sample.

SI Figure 4a: Raman spectra for GcSi-1 painted surface.

SI Figure 4b: Raman spectra for GcSi-1 rock substrate.

SI Figure 5: Comparison of XRD spectra GcSi-1 painted side and rock substrate.

SI Figure 6: Low magnification optical micrographs of GcSi-1.

SI Figure 7: Comparison of FeOB particle before and after *in situ* thermal experiment.

SI Figure 8: SEM micrographs of *L. ochracea* at 600°C, 950°C, and 1050°C during *in situ* heating.

SI Figure 9: Untreated FeOB control sample prepared for *in situ* SEM heating experiment.

SI Figure 10: Series of micrographs showing the use of FIB-SEM to prepare FeOB 800°C control sample for HRTEM analysis.

SI Figure 11: Details of *L. ochracea* sheath morphology.

SI Figure 12: ATR-FTIR spectra of FeOB control samples and GcSi-1.

SI Table 1: Summary of mineral phases identified in GcSi-1 and heat treated FeOB control samples.

**Supplementary Information References Cited**

**---**

**Titles and Captions for Other Supplementary Materials**

SI Data 1: XRD – Raman – ATR-FTIR spectra files.

Link: https://figshare.com/s/c1fa8288391a212c6c5a

SI Data 2: Summary of temperature ramp rates for *in situ* heating.

Link: https://figshare.com/s/0e975ff61db99cba8367

SI Data 3: SQUID magnetometry data.

Link: https://figshare.com/s/006fb5880e4e9c92c833

SI Video 1: SEM *in situ* heating procedure. See SI Text 3b for observational notes.

Link: <https://figshare.com/s/e6337b7466ba779f826b>

SI Video 2: SEM *in situ* heating procedure, holding at 1,050°C.

Link: <https://figshare.com/s/28df08be5029d311e4bb>

SI Video 3: Example of sample preparation using FIB-SEM.

Link: https://figshare.com/s/fde9b70eabc4f9d729e8

**SI Text 1: Overview of Rock Art, Archaeology, and Geology at Babine Lake, British Columbia, Canada**

At a regional scale, clusters of rock art (pictographs) dot the landscape of the Pacific Northwest. Despite a long and demonstrated history of ochre use, mineral pigment preparation for rock art in North America is poorly understood. Rock art research has traditionally focused on interpreting glyphs and imagery [^1^](#_ENREF_1)^,^[^2^](#_ENREF_2), with lesser regard for the paints and how they were produced, although see Wainwright [^3^](#_ENREF_3). They are described by descendant communities and in ethnohistoric records as important communication tools for stories about spiritual beings, and places and events of historical significance [^1^](#_ENREF_1)^,^[^2^](#_ENREF_2)^,^[^4-7^](#_ENREF_4). We focus here on one pictograph panel, Boling Point (GcSi-1), located on the south arm of Babine Lake in the northern Interior Plateau region of British Columbia, Canada. While the temporal sequence of rock art production at Babine Lake is unknown, human occupation in the region dates as early as 5,000 years BP [^8^](#_ENREF_8). At present there are six recorded pictograph panels on Babine Lake, including Boling Point, for a combined amount of >150 individual monochrome glyphs. See SI Fig. 1a-1h for examples of rock art glyphs at multiple sites on Babine Lake. The panels at Boling Point were recorded during a field survey by Mohs and Mohs [^9^](#_ENREF_9), although no further research was conducted until the present study. The glyph imagery on the panel is a combination of figurative anthropomorphic and geometric figures all painted in red pigment, shown in Fig. 1 (main text).

The pictograph specimen examined in this study was collected during the 1976 survey, subsequently archived in the collections at the Royal British Columbia Museum (Victoria, BC), and was analyzed with permission from Babine Lake Nation. The fragment shows two red streaks that intersect on a perpendicular angle (see Fig. 1c, main text). The field notes recorded by Mohs and Mohs include brief descriptions of the pictograph panels, color photographs, and hand drawings, although no interpretive information on glyph imagery is provided. It is unclear from which precise location the fragment was obtained or how it was removed, whether naturally spalled and recovered from the base of the panel or deliberately detached, although likely the latter as this was a common practice of that time. We emphasize here that this method of large bulk sample removal is no longer standard practice today.

Babine Lake is situated equidistant to the Skeena (which it flows into) and Fraser Rivers, two major waterways that run through north interior British Columbia. As such, Babine Lake is a historically-significant node for regional-scale socioeconomic interaction. This nexus connected Pacific Coastal communities to the Interior Plateau by way southwest to the Bella Coola Valley (Nuxalk, Heiltsuk), south and east to Plateau and Fraser Valley communities, north through extensive trail systems (including to the key obsidian source at Mt. Edziza), and north-west along the Skeena River to Tsimshian-speaking groups on the North Coast [^10-12^](#_ENREF_10). Archaeological evidence indicates that this area was an important locality in Coast-Interior exchange networks [^13^](#_ENREF_13)^,^[^14^](#_ENREF_14). Ethnohistoric documentation makes brief reference to ochre pigment as a common exchange item between Coastal and Interior (Carrier group) communities along this route [^15^](#_ENREF_15), though no ochre provenance study has been carried out to date. Despite the inherent value of ethnographic evidence, it does leave our technological questions regarding the selection, preparation, and application of ochre mineral pigments unanswered.

The history of indigenous settlement on Babine Lake is currently under investigation as part of a long-term community-engaged archaeological project. Preliminary results corroborate oral histories and historic accounts on subsistence economic practices. Ethnohistoric records [^11^](#_ENREF_11) indicate that Babine Peoples harvested large volumes of Skeena River salmon with the use of wooden fish weirs, but the antiquity of this practice was unknown. Recent investigations have revealed a large fishing village dating to at least 1,300 years before present [^13^](#_ENREF_13), and preserved fish weir elements daring to 1,000 years ago [^16^](#_ENREF_16).

The geology in the area of Babine Lake is characterized by a complex succession of Paleozoic carbonates, island-arc volcanic and volcanoclastic rocks, basaltic volcanics, and marine clastic sedimentary rocks[^17^](#_ENREF_17). Most of the rock art panels on Babine Lake are painted on large, prominent outcrops of basaltic or andesitic volcanics, with the exception of Boling Point, being a large outcrop of lower Permian massive grey argillaceous limestone.

**SI Text 2: FeOB habitat, biogeochemistry; sampling protocol for *L. ochracea* controls**

Fe-oxidizing bacteria (FeOB) are a group of biomineralizing chemolithotrophic organisms that proliferate in acidophilic or neutrophilic aquatic habitats, and their remnant microfossils have taxonomic features that enable their identification at the genus and species levels. They play an important role in biogeochemical cycling of metals on Earth, as well as in our understanding of banded iron formations and deep-sea environments [^18-21^](#_ENREF_18) and the role of cyanobacteria in early life on planet Earth [^22^](#_ENREF_22). Freshwater groups are generally classified into three main genera: *Gallionella* and *Sideroxydans* are chemolithotrophs while *Leptothrix* is a mixotroph [^23^](#_ENREF_23)^,^[^24^](#_ENREF_24). Our focus here is on the neutrophilic type, which form highly structured yet delicate microbial mats in aquatic environments. They proliferate by oxidizing ferrous ions to ferrous oxides, and precipitate distinctive sheath- or stalk-like structures of Fe-oxyhydroxides as a function of their metabolic cellular reproduction [^25^](#_ENREF_25). Basic requirements for FeOB growth are circumneutral pH environment and a constant source of Fe(II) coupled with low oxygen [^26^](#_ENREF_26). The sheath structures, most often found empty due to cellular autolysis, exhibit some degree of microfossil preservation in nature under ideal conditions [^27^](#_ENREF_27). It is common for multiple FeOB genera or species to co-occur in a single microenvironment, with one predominant species.

*L. ochracea* grow distinctive colonies of micro-tubular sheaths, typically ~1μm in diameter and 5-10 μm in length. The microfossils are produced by single cells that continuously grow in a chain-like formation at the distal end, leaving behind a Fe-oxide biomineralized filament [^28^](#_ENREF_28). The basal end is attached to a substrate, often observed as siliciclastic material (quartz sediment grains). The sheaths are composed of a complex hybrid of organic and inorganic materials consisting of an exo-polysaccharide core surrounded by a highly thermostable Fe:Si:P nanocomposite (roughly 73:22:5), with sorbed or structural impurities of C, OH, PO_4_, SO_4_, and Ca [^23^](#_ENREF_23)^,^[^25^](#_ENREF_25)^,^[^29^](#_ENREF_29). At maturity, the sheaths exhibit a fibrous outer coat, with some examples having double-walled inner rings [^30^](#_ENREF_30) (see Figs. 3g and 3i). The resulting formation resembles a microbial mat, typically orange-brown in color, which grows on the margins of ponds, streambeds, and springs. The samples evaluated here were collected from a small first-order stream sourced from a beaver point. Shallow (1-2 cm thick) ocherous iron mats covered approximately a square meter in a slowly flowing segment of the stream. Water temperature at the time of sampling (October 2017) was 12°C, the pH was 6.5, and the Fe(II) concentration approximately 40 µM. The mat material was collected using a 10 ml pipette with care taken to sample only the upper surface of the mat, and shipped on ice to the University of Missouri.

**SI Text 3: Notes on *in situ* Videos**

*3a: FIB-SEM Sample Preparation*

Our use of the Protochip Arduro was non-traditional and sample preparation proved challenging. Affixing a suitably-sized *L. ochracea-*enriched sediment particle onto the heat-activation area and ensuring it would remain in place while coupling the device to the SEM and pumping down the chamber to the operational pressure was problematic and time-intensive. Once the heating experiment was underway it would be impossible to move or reorient the sample if needed. Therefore, to optimize our ability to observe structural changes during heating, it was necessary to locate a single grain of *L. ochracea*-enriched sediment that would ideally show a representative cluster of sheaths oriented in multiple directions. To prepare the sample we improvised a creative solution, taking advantage of access to a FEI SCIOS FIB SEM instrument. We deposited a small quantity of the untreated FeOB-enriched sediment onto a TEM grid. Using the FEI SCIOS FIB SEM instrument, we scanned the sediment grains and selected the ideal sample. We navigated a nano-manipulator needle to the grain and secured it to the grain with a Pt spot-weld. Once secure, we lifted the sample grain and deposited it onto the heat-activation area of the Protochip, taking care not to compromise a critical thin membrane on the surface of the chip. The grain was secured to the chip with additional Pt spot-welds, and a Ga ion beam was used to detach the needle tip. SI Fig. 9 shows the prepared FeOB-enriched particle with *L. ochracea* sheaths, diatoms, and other features. SI Video 3 is a recording of the procedure. With the sediment sample mounted in place on the Protochip, we proceeded to perform the heating experiment in a FEI Quanta 600 FEG Environmental SEM, operating in low vacuum mode.

FIB-SEM was similarly used to prepare a cross section of one of each of the GcSi-1 and FeOB 800°C samples for HRTEM analysis. SI Fig. 10 shows features of the FeOB 800°C sample preparation.

*3b: Observational Notes on in situ heating procedure*

Private link to video on Figshare repository: https://figshare.com/s/e6337b7466ba779f826b

The *in situ* heating procedure took place over 4h 8m. Temperature was ramped at a rate of 0.5°C per second, with intermittent holds at 500°C, 600°C, 700°C, 800°C, 900°C, 950°C, and 1,050°C to capture SEM micrographs. SI Data 2 is a summary of temperature ramp rates and holds for the duration of the experiment. Our intended goal was to reach the maximum temperature capability of the Protochip (1,200°C), however, the chip membrane combusted and failed at 1,142°C, leaving the FeOB control sample unrecoverable. Fortunately, we were able to capture a significant amount of valuable information leading up to the point of Protochip failure.

Temperature was held at 500°C, 600°C, 700°C, 800°C, 900°C, 950°C, and 1,050°C to capture SEM micrographs as we observed key melt-phase and morphological changes. The greatest change we observed between ambient temperature and 500°C was the shrinkage of the entire FeOB-enriched sediment due to structural OH loss. Between 600 and 700°C, there was significant slumping and warping of the sheath structures throughout. In the range of 700–800°C, the sheaths exhibited the appearance of “sweating” or “beading”, as Fe:Si phase separation begins to occur. Above 800°C, the nucleation of crystalline polymorphs (hematite, magnetite, and silica microspheres) on the outer surfaces of the sheaths rapidly proliferated. A diatom fragment (lower center) began to slump and melt, and as temperature approached 900°C, significant sheath degradation began to occur. Between 900-950°C, the sheath structures appeared to degrade and continue to warp, and slump. By 950°C, the diatom had nearly completely melted, and hematite microspheres began to coalesce into larger conglomerates. Between 950°C and 1,000°C, hematite and magnetite formation continued to nucleate as the sheaths degraded, and tetra- and octahedral polymorphs were produced. SI Figure 8 shows a cluster of sheaths at 600°C, 950°C, and 1,050°C, illustrating the increase in phase separation and sheath degradation. Note the crystallization of angular polymorphs of hematite and magnetite at 1,050°C. At 1,050°C, the Pt-weld holding the FeOB particle in place began to melt leading subsequent failure of the Protochip. SI Video 3 is footage of a hold at 1,050°C, highlighting the new hematite phase nucleation and separation and recrystallization of amorphous SiO_2_.

*3c: Comparison of Furnace Heating vs. Protochip Heating*

There are important considerations when comparing the observations and between furnace and Protochip heating. The temperature ramp rates, holds, and heating environments differed between the two heating procedures. The furnace heating ramped at a rate of 100°C per hour, held for 3 hr, then cooled slowly to ambient temperature in an oxygenated environment. By comparison, the *in situ* procedure ramped at a more rapid rate of 0.5°C per second, with multiple holds at 500°C, 600°C, 700°C, 800°C, 900°C, 950°C, and 1,050°C for varying lengths of time ranging between ~10-77 minutes. The *in situ* heating was a comparatively reduced oxygen environment (due to water vapor at 40 Pa), which may have resulted in a lag inhibiting the nucleation of new Fe-oxide phases. Furthermore, because it was not possible to evaluate crystallization of high temperature Fe-oxide polymorphs as after peak temperature was reached, we do not know if larger crystals would have formed upon cooling for the *in situ* heating example.

SI Fig. 11 is a detail comparison of *L. ochracea* sheath morphology at high temperatures for furnace heating (1,000°C), and *in situ* heating (1,050°C), respectively. The images were captured either after the sample cooled, as in the case of the furnace-heated example, or, live during the heating procedure, as for the *in situ* example. Differences in temperature and heating environment may account for some of the variation observed. For both, hematite and magnetite formation were evident, though their distribution differed. In the furnace-heated controls, hematite and magnetite phase formation appeared to be restricted to the exterior surfaces of the sheath bodies, with remnants of the tubular structure shapes remaining relatively intact. Whereas, in the *in situ* reduced oxygen environment, the phase separation and recrystallization appeared more robust and complete. It is likely that the lower pressure environment in the SEM contributed to minor shifts in the temperature at which phase changes occurred. It is also likely that slower cooling and increased oxygen in the furnace environment could have further induced conglomeration of the individual Fe-oxide polymorphs into larger particle sizes. When we consider other archaeological evidence for firing methods typically used by the inhabitants of Babine Lake (high temperature open domestic hearth, or low temperature pit hearth), it is conceivable that the oxygenated muffle furnace control samples would be a more accurate proxy for open hearth firing conditions.

**SI Text 4: Expanded Description of FeOB XRD data**

Identifying characteristic peak patterns in high-purity, crystalline materials can be straightforward. But, mineralogically heterogeneous matrices are challenging to interpret largely due to spectral interferences. Many of the Fe-oxide/oxyhydroxide phases of interest for this study are poorly ordered and stoichoimetrically complex, resulting in features of peak broadening and increased uncertainty in phase identification. Moreover, the (untreated) structures of *L. ochracea* biominerals are a complex inorganic-organic hybrid material, non-crystalline and often undetectable by XRD [^30^](#_ENREF_30). Nonetheless, we were successful in identifying trends in mineral phase transformations at increasing thermal gradient.

Quartz is dominant in all FeOB control samples regardless of temperature. At temperatures at and below 600°C, iron phosphates and iron hydroxides contribute much of the low abundance peaks and spectral noise below ~10°Θ, while above 600°C they are low abundance or no longer detectable. Intermediate phases of Fe-oxides, including FeO and lepidocrocite appear between 200°C and 600°C. Magnetite and hematite do not form until 400°C and 600°C, respectively, and iron silicates only occur at 800°C and 1,000°C. In its natural form, iron silicate is an end member of olivine, with the mineral name fayalite [^31^](#_ENREF_31). We attribute its presence in the GcSi-1 sample to melt and recrystallization. In studies related to the formation of fayalite in silicon-containing steels, the presence of trace amounts of phosphorous (0.115 wt. % P_2_O_5_), reduces the temperature at which fayalite forms from 1,173^o^C to 954.2 ^o^C, and is further reduced in the presence of increasing concentrations of phosphorous, observed as low as 890^o^C [^32^](#_ENREF_32).

Although most major peak patterns were successfully attributed to a phase, some minor peaks remain unassigned, especially in the low and intermediate temperature examples. We attribute this in part to incomplete combustion of unidentified organic materials, partial crystallization of new phases, and the possible presence of poorly ordered ferric phosphates, calcite or clay particle aggregates, calcium silicates, or pyroxenes. With a focus on key transitions in iron oxide phase change, the results observed in the XRD patterns are consistent with our expectations for temperature-induced phase transitions in *L. ochracea*–enriched sediment.

**SI Text 5: SQUID Magnetometry**

SQUID magnetometry is a technique that uses high sensitivity instrumentation to detect subtle magnetic fields. Magnetic hysteresis measurements were performed on a Quantum Design Magnetic Properties Measurement System (MPMS). The FeOB samples were loaded into gelatin capsules inside a plastic straw with empty capsules above and below to reduce the background contribution from gelatin. Magnetization at 300 K was measured as a function of applied field in the range of +/- 20 kOe and normalized to sample mass. The graphical outputs of the measurements are a series of hysteresis loops, shown in Fig. 8. The magnetic measurements show that the untreated and 200°C FeOB samples are paramagnetic at room temperature. The 400°C and 600°C samples both show magnetic saturation at less than 3 kOe applied field, with no hysteresis, consistent with superparamagnetic Fe_2_O_3_ nanoparticles. This is consistent with our observation of initial formation of Fe:Si phase separation and recrystallization at ~600°C via SEM examination. Recall from the SEM observations that at 800°C, the Fe-oxide particles begin to coalesce into larger particles. This is further evidenced by a sharper saturation curve and by the appearance of hysteresis in the magnetic measurement of the sample as the particles become large enough to display weak ferromagnetism. Finally, at 1,000°C, the saturation magnetization of the sample drops by an order of magnitude. Overall, our results are consistent with other reports that Fe-oxides heated to at least 750°C to 800°C exhibit characteristics of superparamagnetism [^33^](#_ENREF_33).

**SI Text 6: ATR-FTIR Analysis**

ATR-FTIR (attenuated total reflection – Fourier transform infra-red) spectroscopy was attempted as a complementary method to evaluate inorganic mineral phase transformations and to determine if any organic components of the FeOB-enriched sediment could be identified. Sub-samples of the GcSi-1 and FeOB controls were each homogenized in a clean agate mortar and pestle, pressed into KBr pellets and analyzed by ATR-FTIR using a Nicolet 4700 FT-IR mid-infrared instrument. The targeted spectral range was 400-4,000 cm^-1^ wavenumbers. Spectral features were identified using a combination of reference spectra from the RRUFF database and Namduri and Nasrazadani [^34^](#_ENREF_34).

Our interpretations of the ATR-FTIR spectra were limited by spectral interferences. SI Fig. 12 shows the ATR-FTIR spectra and summarizes band regions in the fingerprint region. The functional group region (4,000 cm^-1^ to 1,450 cm^-1^) is where features characteristic of stretching vibrations of major functional groups (i.e. C-H, N-H, etc.) will appear, while the fingerprint region (1,450 cm^-1^ to 500 cm^-1^) shows bending vibrations within the molecule. O-H stretching bands in the functional group region at ~3,400 cm^-1^, and in the fingerprint region at ~1,640 cm^-1^, attributed to H_2_O, are present in all samples from untreated to 1,000°C, however with decreased intensity as temperature gradient increases. However, the ~1640 cm^-1^ peak could also be attributed to a N-H stretch representative of either a saccharide compound produced by the *L. ochracea*, or a secondary amine (acetyl group, saccharide) or amide (amino acid), or both. The peaks at ~2,900 cm^-1^, in low abundance in the untreated sample, though clear in all other samples, may be attributable to C-H stretches representative of organic materials. However, most functional group peak assignments remain ambiguous in large part due to spectral interferences in the fingerprint region caused by the abundance of quartz.

As the major impurity in the FeOB control samples, quartz dominated the spectra with strong absorbance in the same fingerprint region as some of the iron oxide phases of interest. Strong peaks for quartz were observed in all spectra (untreated to 1,000°C), between 1,000–1,200 cm^-1^, 775-795 cm^-1^, 690 cm^-1^ - 695 cm^-1^, and 430-460 cm^-1^, respectively. Calcite, as part of the rock substrate, was identified in GcSi-1 at 1,419 cm^-1^, 875 cm^-1^, and 713 cm^-1^, and was expectedly absent in the FeOB controls. In all samples quartz dominated the spectra with large spectral features at ~460 cm^-1^, in the same region where hematite (470 cm^-1^ and 540 cm^-1^), and magnetite (570 cm^-1^ and 400 cm^-1^) occur. Due to the spectral interferences from quartz and calcite, we could not reliably or confidently assign many spectral features to the presence of iron oxide phases.

**Section: SI Figures**

**
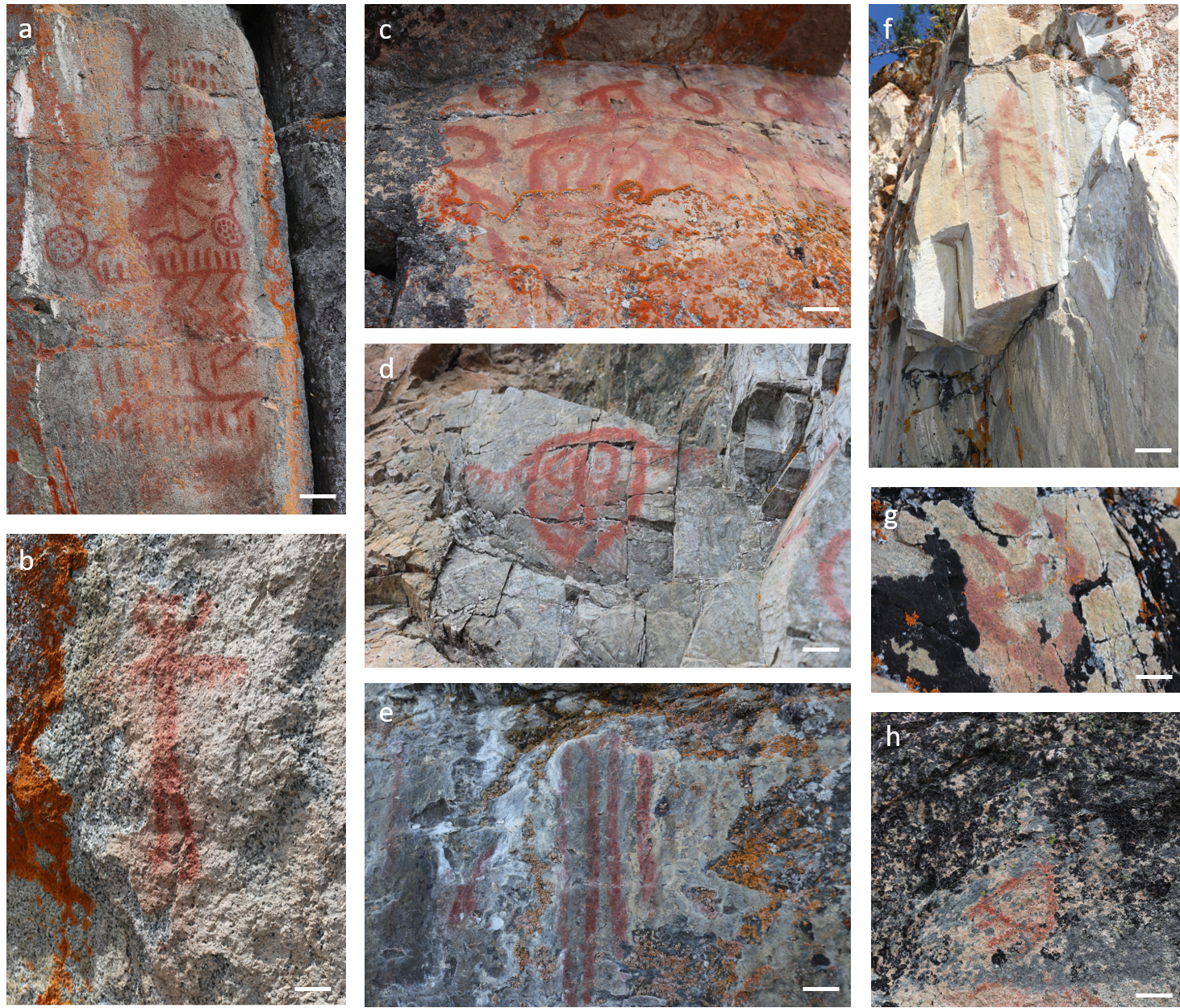
**

**SI Figure 1: Selected images of Babine Lake rock art**. (a) A complex panel showing a large animal figure, a fish, a series of dots and tally marks, zigzag lines, and circles with dots inside. Scale bar = 5 cm. (b) Standing figure with wings, interpreted as a ‘transformer’ mythological being. Scale bar = 5 cm. (c) a panel of circles and lines. Scale bar = 3 cm. (d) Animal face with antlers. Scale bar = 3 cm. (e) Four vertical lines. Scale bar = 3 cm.(f) Bird figure. Scale bar = 3 cm. (g and h) Bear claws. Scale bar = 3 cm.


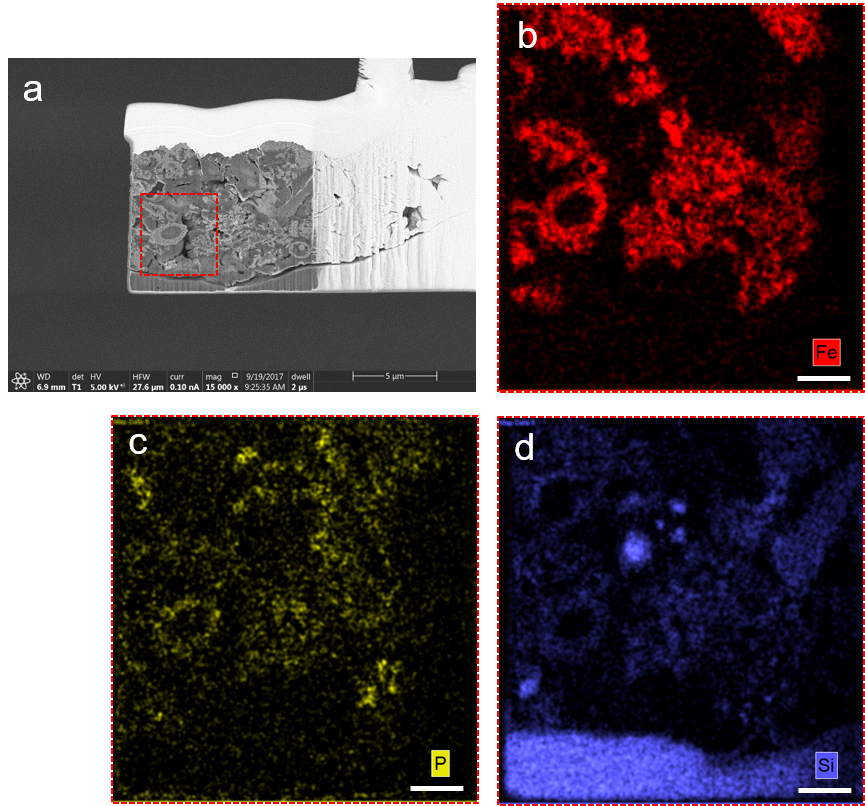


**SI Figure 2: SEM-EDS hyperspectral maps of GcSi-1**. (a) micrograph of lamella cross section prepared for TEM. (b-d) EDS maps of iron, phosphorus, and silicon, illustrating major phases present in sheath body matrix. Scale bar = 2.5 µm.


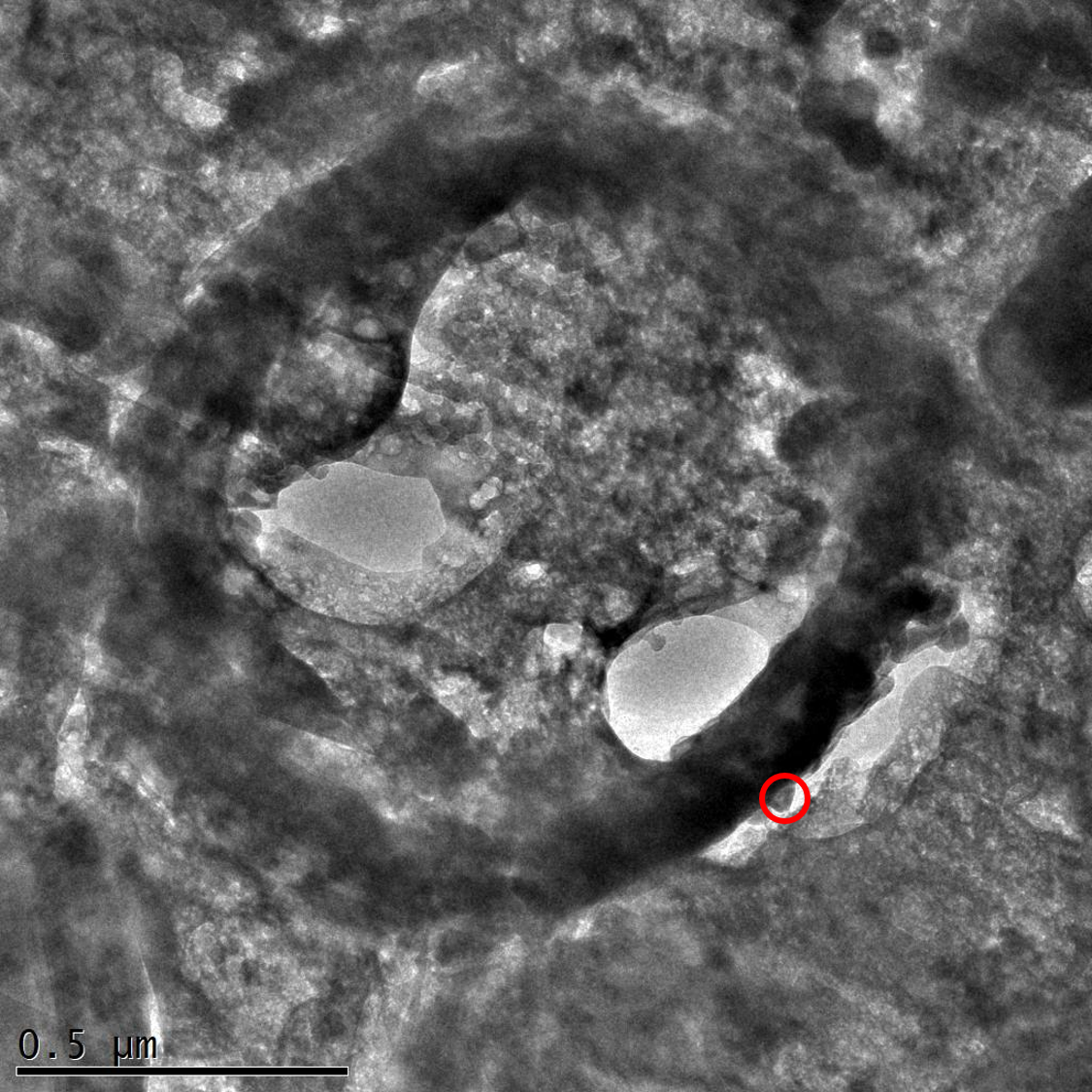


**SI Figure 3a: TEM image of a cross-sectional lamella from GcSi-1 sample, prepared for HRTEM analysis.** The circled region indicates where Fig. 5 (main text) and SI Fig. 3b were taken.

**
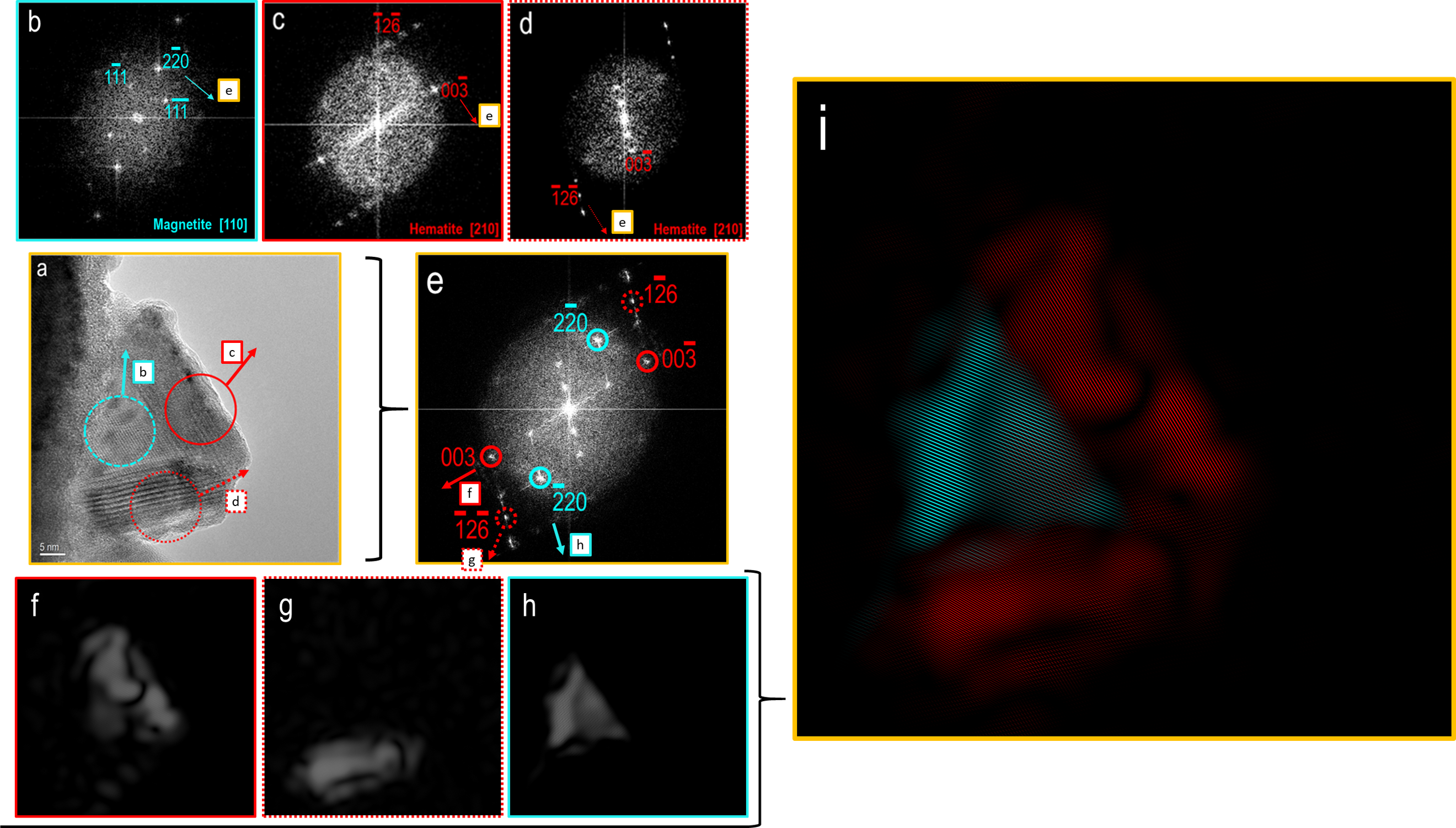
**

**SI Figure 3b: Localized hematite and magnetite nanoparticle precipitated on the surface of a *L. ochracea* sheath in the GcSi-1 sample.** (a) HRTEM image showing the magnetite particle (indicated by the cyan circle) coated with hematite phase (indicated by the red circles). (b) Fast Fourier Transform (FFT) pattern from the region by the cyan circle in (a). It can be well indexed based on a magnetite phase (Inorganic Crystal Structure Database #65339) along [110] zone axis. (c) and (d) FFT patterns are from the red circled region and the dotted red circled region in (a) respectively. Both patterns can be indexed based on a hematite phase (Inorganic Crystal Structure Database #161292) along [210] zone axis. (e) Three pairs of diffraction spots were selected from the FFT of the full region as uniquely corresponding to the structures identified in (a-c); {002} pairs was used to generate magnetite (220) lattice fringes in the cyan circled region, {126} pairs for hematite phase (126) lattice fringes in the dotted red circled region and {003} pairs for hematite phase (003) lattice fringes in the red circled region. Masks were generated to isolate these pairs, whereupon an inverse FFT extracted those particular fringes from the original image. The generated images were adjusted and composited to yield Fig. 5 (main text). Note that (00-3) from the hematite phase spot in b is very close to the (2-22) from magnetite phase. However, the intensity of the (2-22) from the magnetite phase is very weak compared to the (00-3) and thus its contribution to the (00-3) lattice fringes is negligible when doing the filtering and inverse FFT using (00-3).


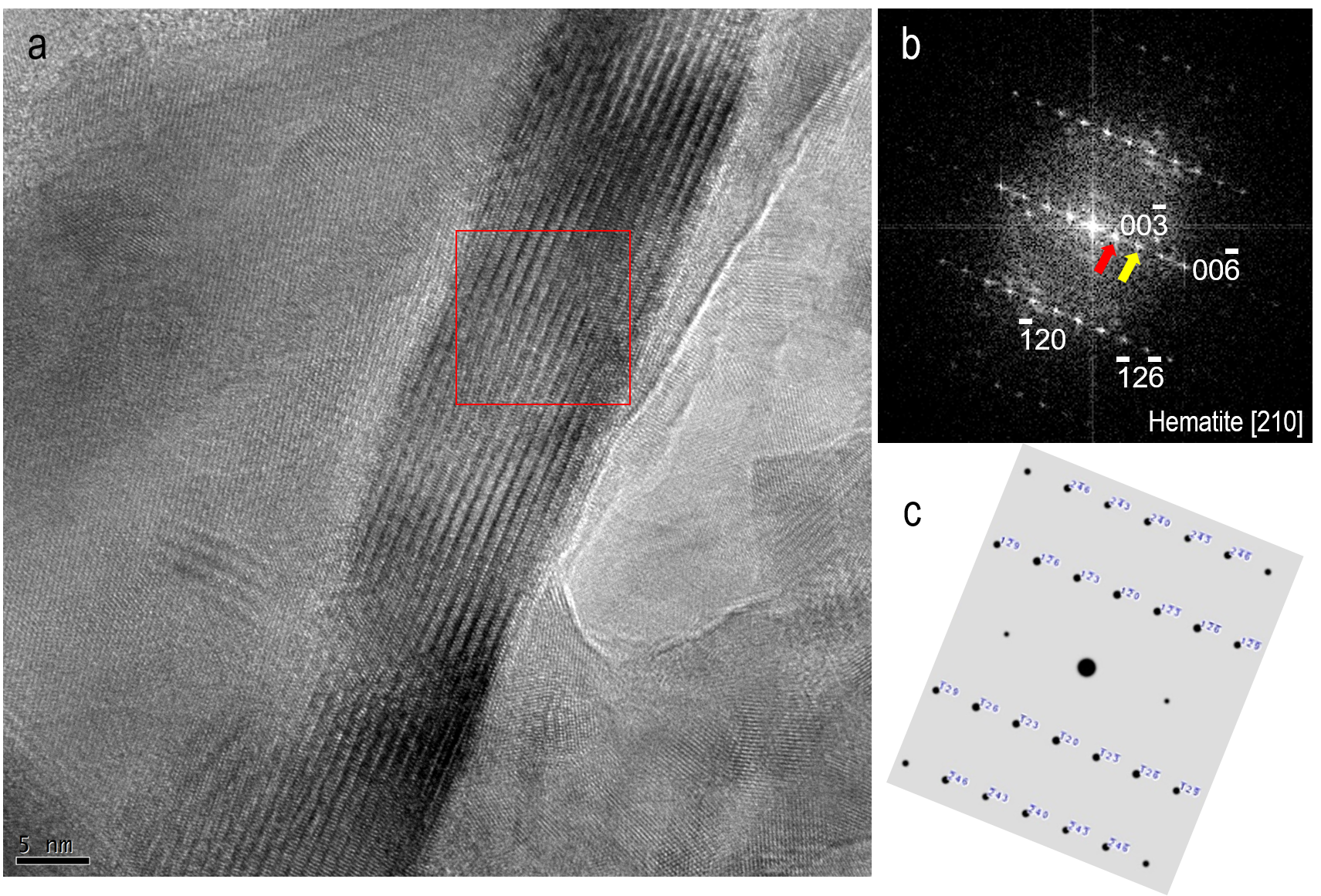


**SI Figure 3c: Localized hematite nanoparticle precipitated on the surface of a *L. ochracea* sheath in the GcSi-1 sample.** (a) HRTEM image showing localized hematite nanoparticle precipitated on the surface of a *L. ochracea* sheath in the GcSi-1 sample. (b) FFT patterns of the region in (a) highlighted by the red square. The pattern can be well indexed based on a hematite phase along [210] zone axis. The (00-3) spot indicated by the yellow arrow re-appears due to the dynamic scattering. The spots indicated by the red arrow that are missing in the simulated pattern in (c) is due to the superstructure that are very likely produced by the oxygen vacancies ordering. The simulated pattern (c) is in good agreement with the FFT pattern (b).


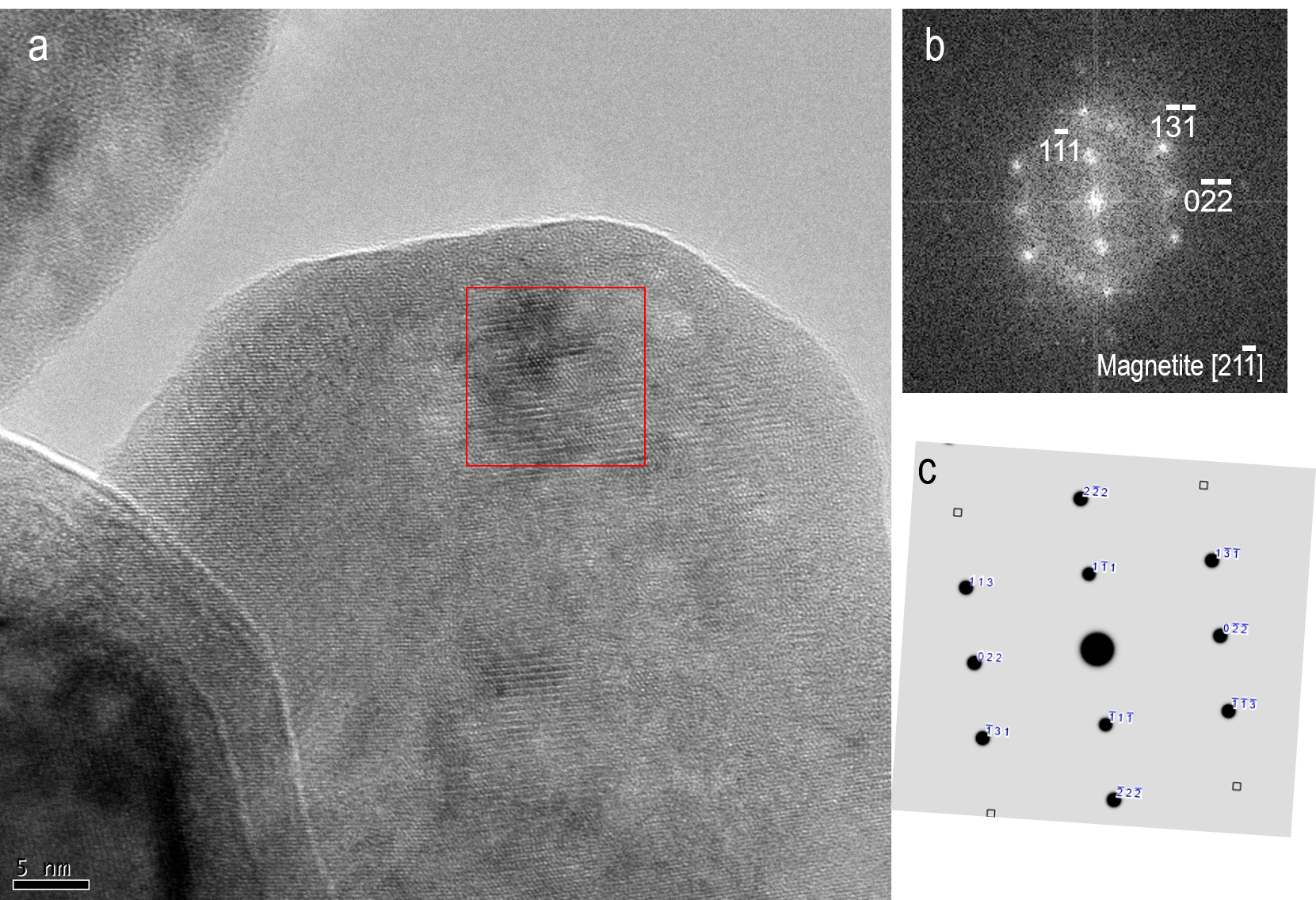


**SI Figure 3d: Localized magnetite nanoparticle precipitated on the surface of a *L. ochracea* sheath in the GcSi-1 sample.** (a) HRTEM image of localized magnetite nanoparticle precipitated on the surface of a *L. ochracea* sheath in the GcSi-1 sample. (b) FFT pattern from the region in (a) highlighted by the red square. The pattern can be well indexed based on a magnetite phase along [211] zone axis. The simulated pattern (c) is in good agreement with the FFT pattern (b).


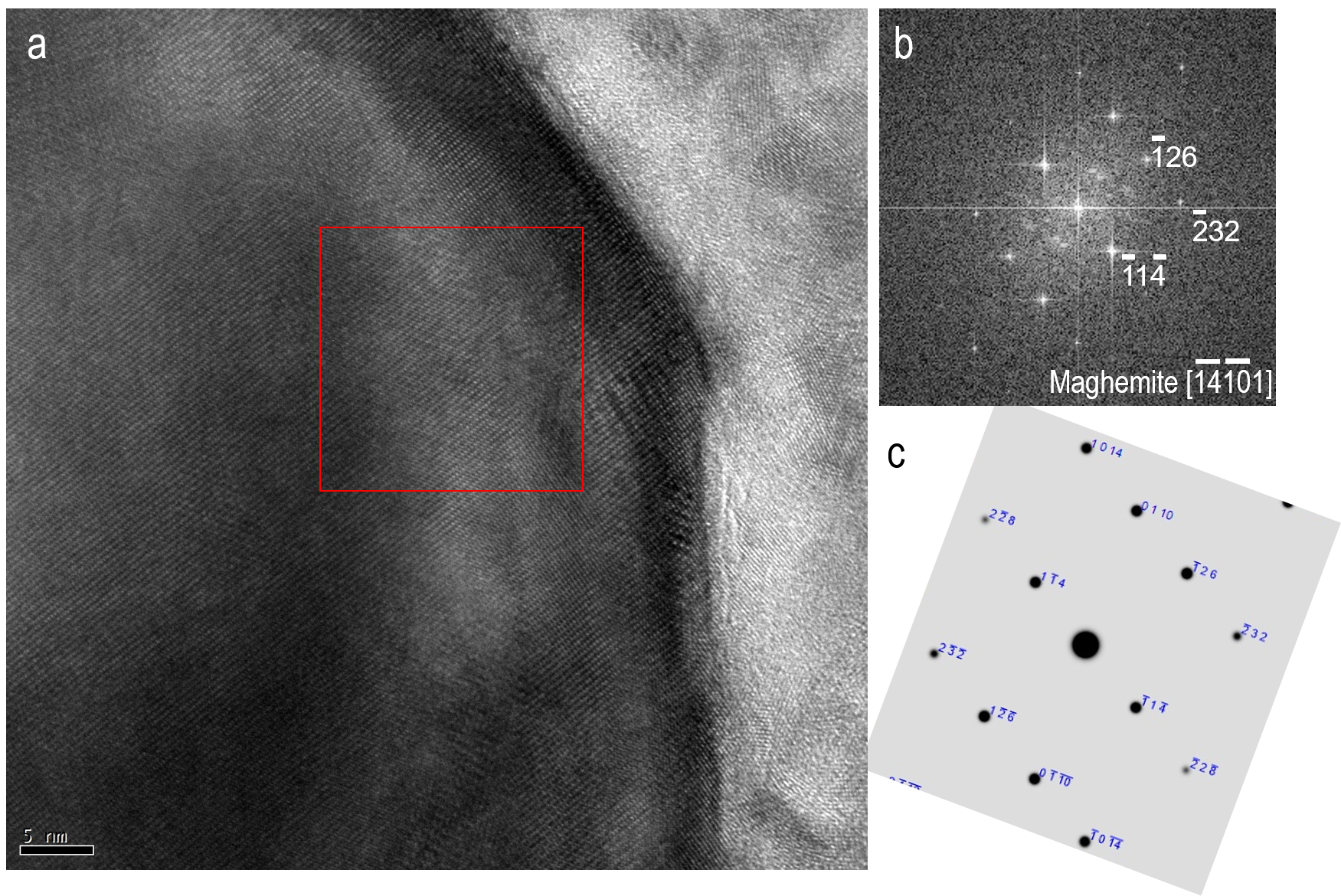


**SI Figure 3e: Localized maghemite nanoparticle precipitated on the surface of a *L. ochracea* sheath in the GcSi-1 sample.** (a) HRTEM image of localized maghemite nanoparticle precipitated on the surface of a *L. ochracea* sheath in the GcSi-1 sample. (b) FFT pattern from the region in (a) highlighted by the red square. The pattern can be well indexed based on a maghemite phase (Inorganic Crystal Structure Database #172906) along [$\overline{14}\overline{10}1$] zone axis. The simulated pattern (c) is in good agreement with the FFT pattern (b).


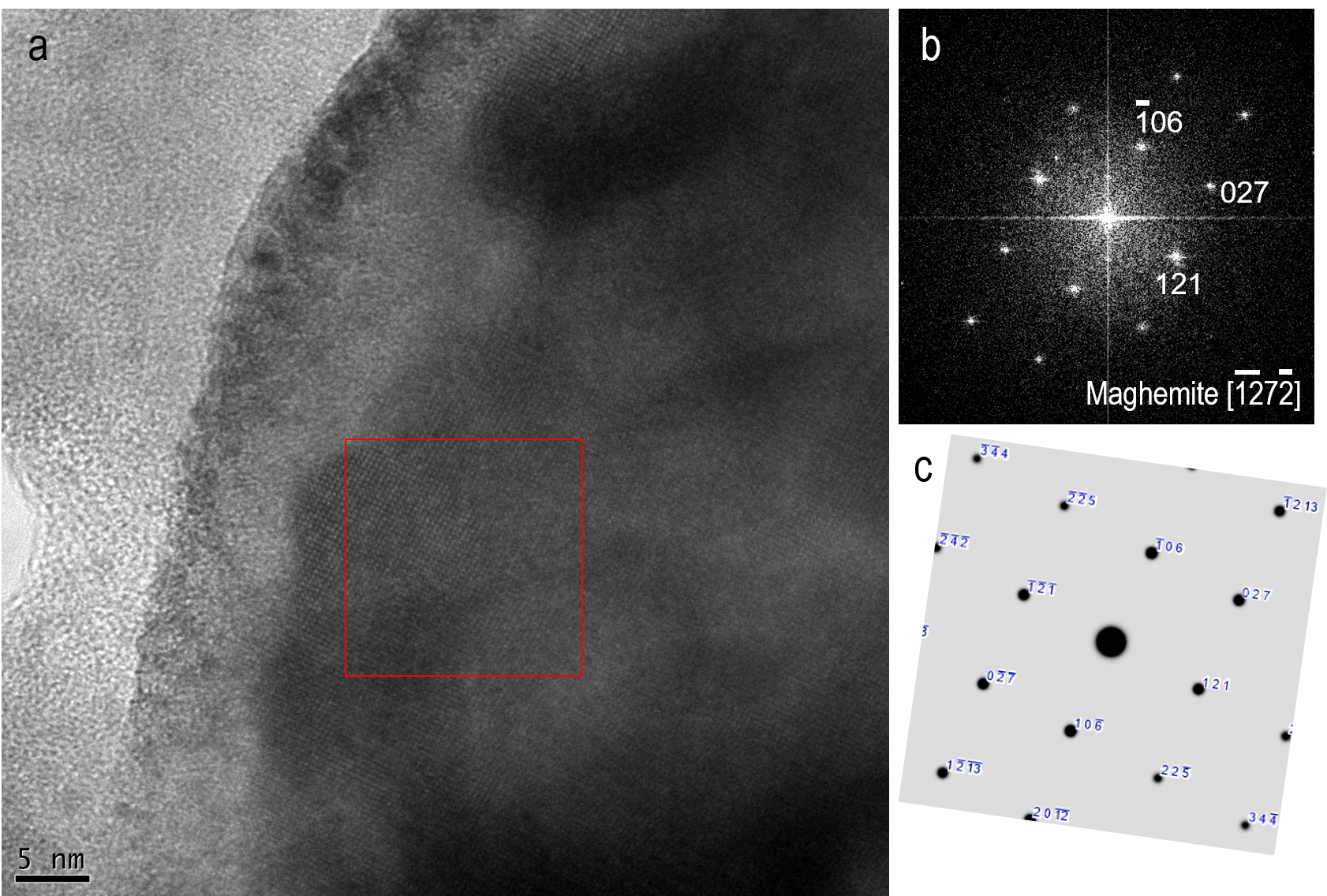


**SI Figure 3f: Localized maghemite nanoparticle precipitated on the surface of a *L. ochracea* sheath in the GcSi-1 sample.** (a) HRTEM image of localized maghemite nanoparticle precipitated on the surface of a *L. ochracea* sheath in the GcSi-1 sample. (b) FFT pattern from the region in (a) highlighted by the red square. The pattern can be well indexed based on a maghemite phase along [$\overline{12}7\overline{2}$] zone axis. The simulated pattern (c) is in good agreement with the FFT pattern (b).

**
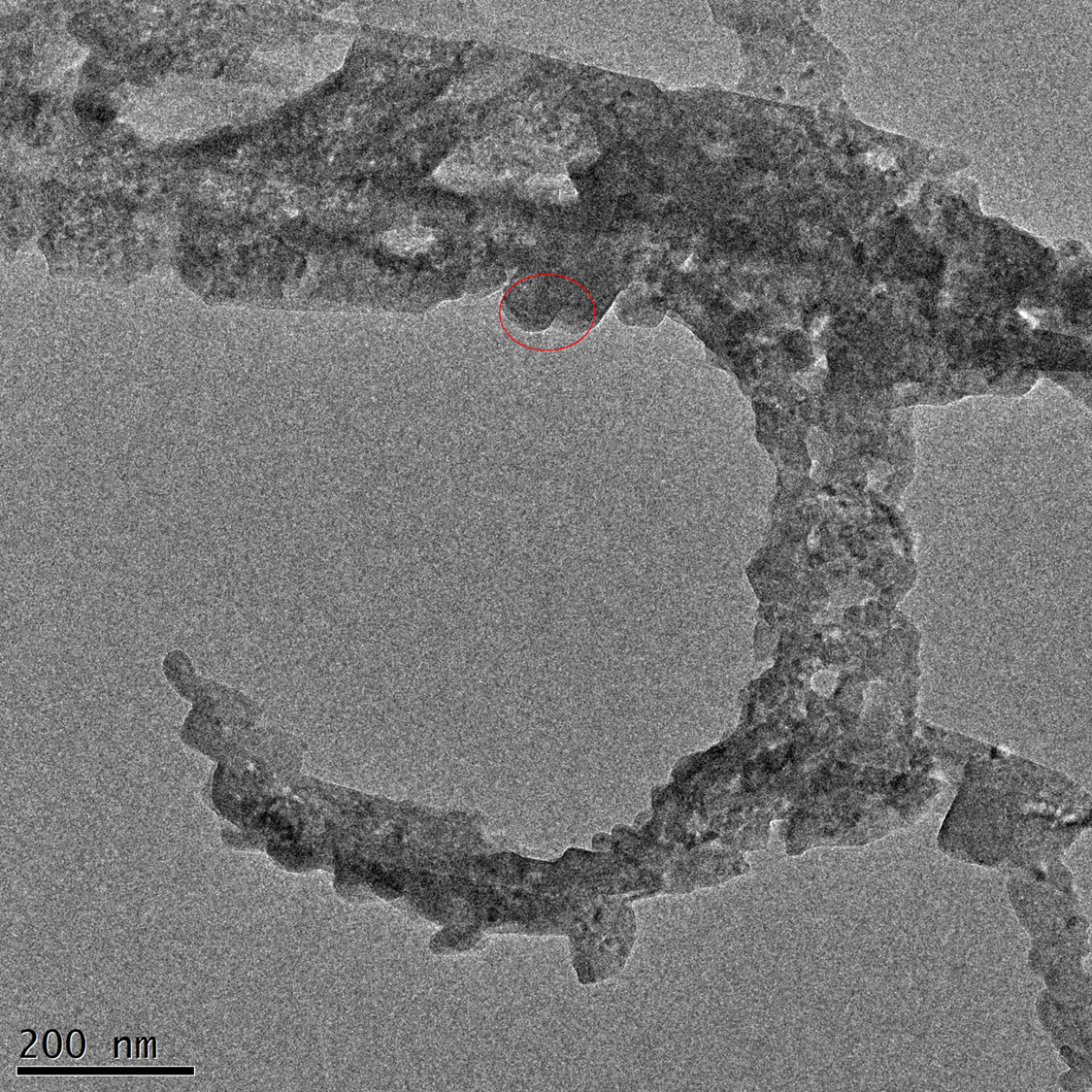
**

**SI Figure 3g: Cross-section of *L. ochracea* sheath from FeOB 800°C control sample prepared for HRTEM analysis.** The red ellipse indicates region of interested targeted in SI Fig. 2h.


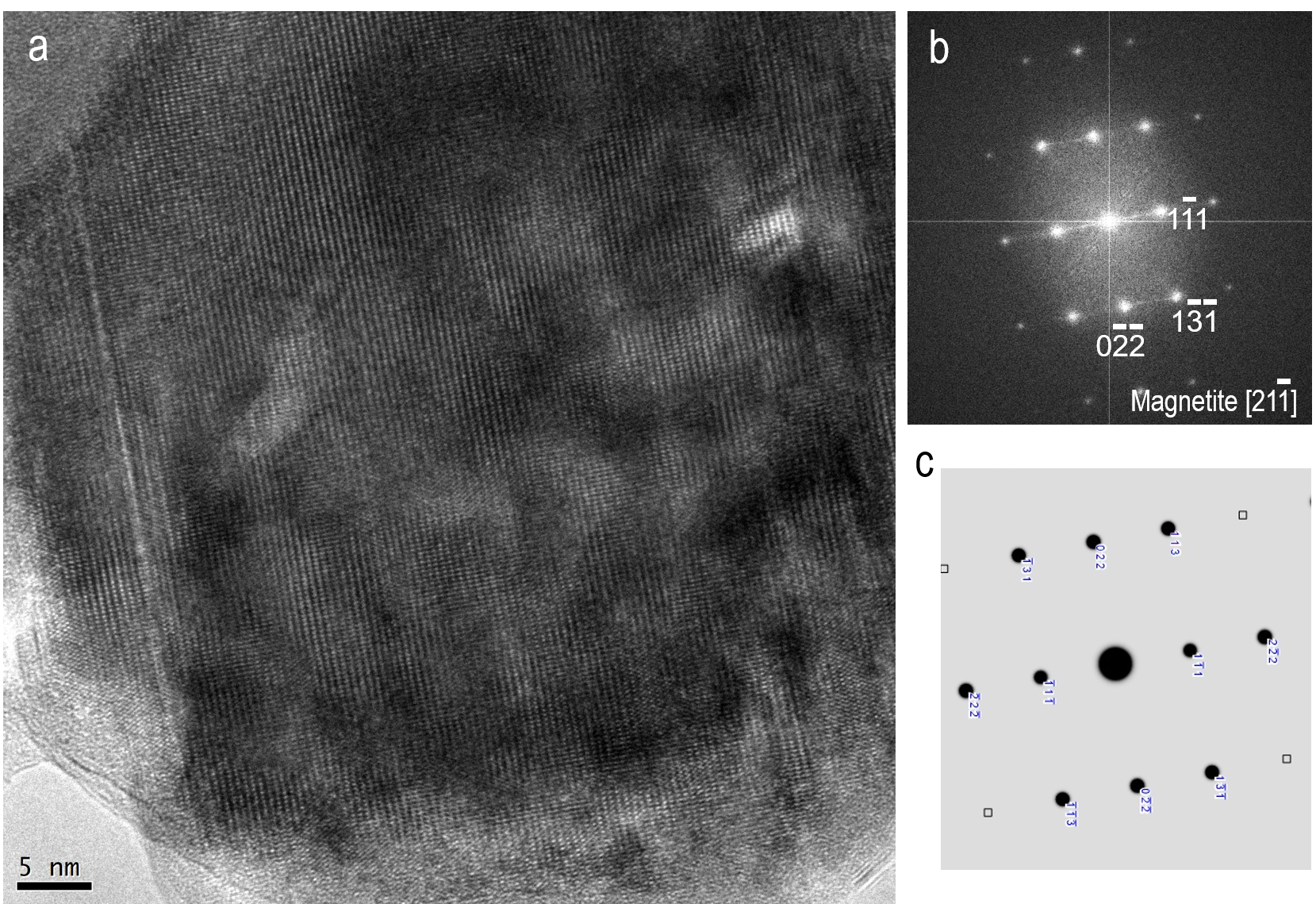


**SI Figure 3h: Localized magnetite nanoparticle precipitated on the surface of a *L. ochracea* sheath in the FeOB 800°C control sample.** (a) HRTEM image of localized maghemite nanoparticle highlighted by the red ellipse in SI Fig. 2g precipitated on the surface of a *L. ochracea* sheath in the GcSi-1 sample. (b) FFT pattern from the (a). The pattern can be well indexed based on a magnetite phase along [$21\overline{1}$ ] zone axis. The simulated pattern (c) is in good agreement with the FFT pattern (b).

**
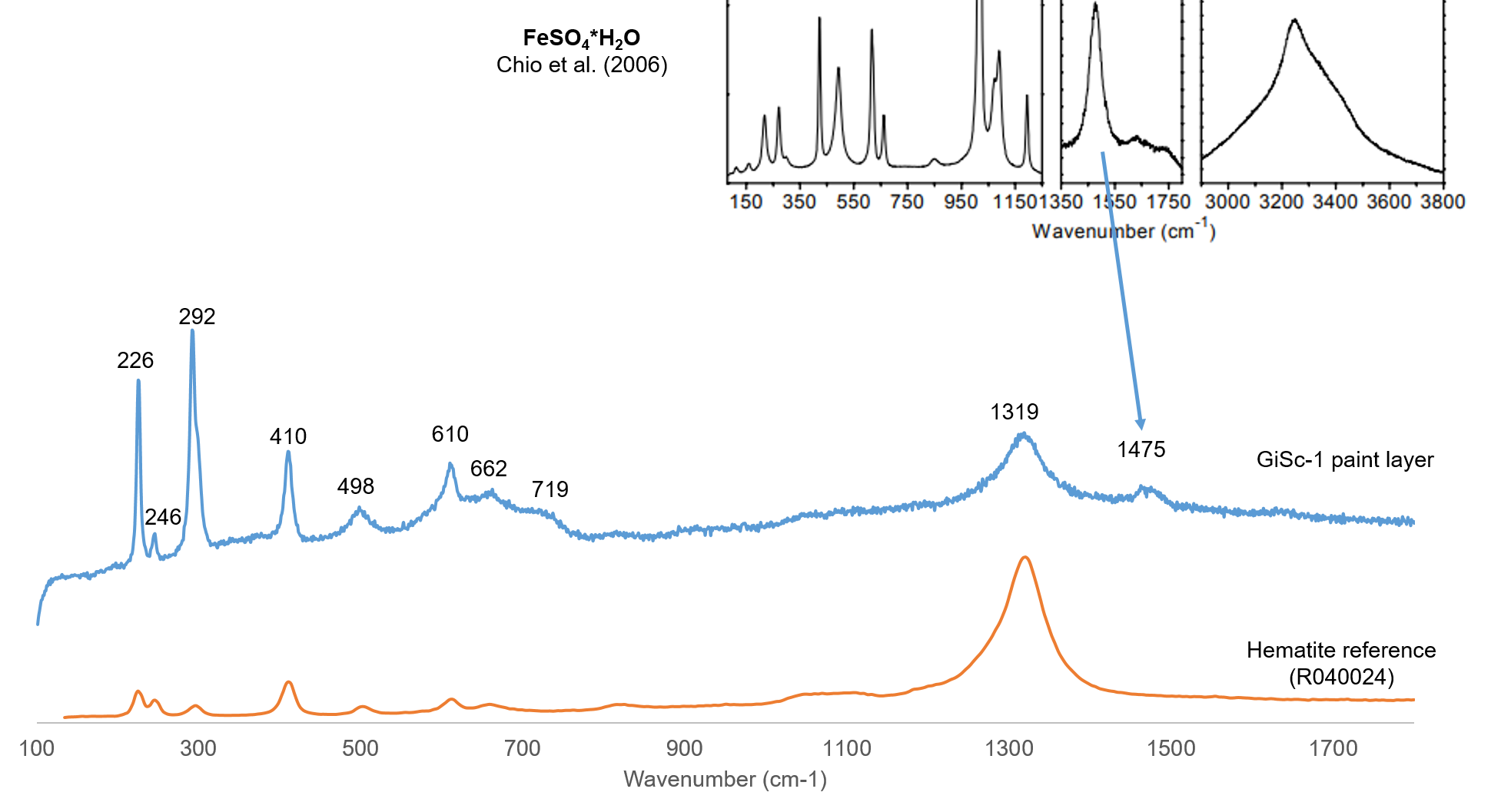
**

**SI Figure 4a: Raman spectra for GcSi-1 painted surface.** The GcSi-1 paint layer shows all characteristic peaks for hematite (Fe_2_O_3_). The peak at wavenumber 1475 cm^-1^ is attributed to the phase FeSO_4_*H_2_O, as identified in Chio et al [^35^](#_ENREF_35). The hematite reference spectrum was downloaded from the RRUFF mineral database (#R040024).

**
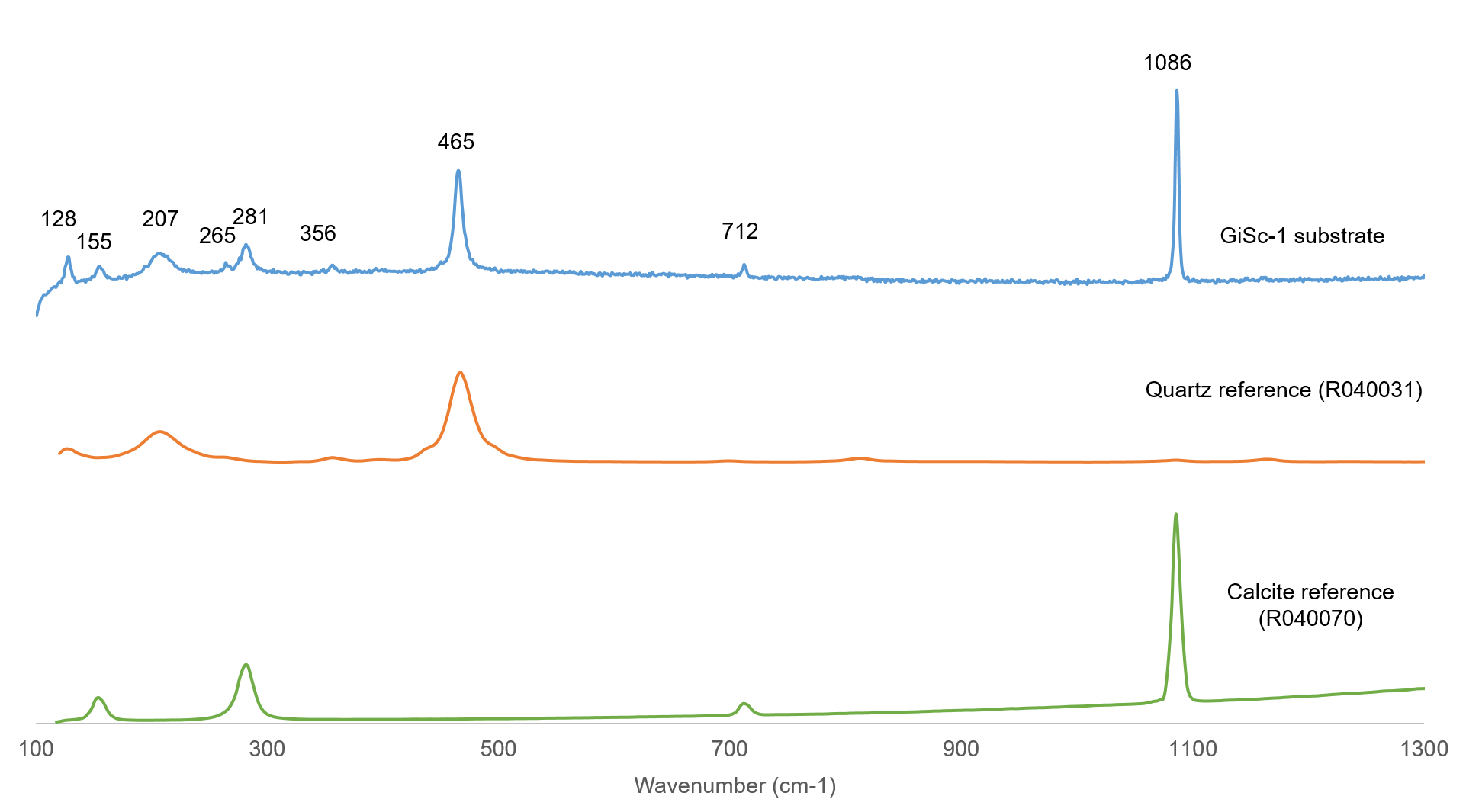
**

**SI Figure 4b: Raman spectra for GcSi-1 rock substrate.** The GcSi-1 rock substrate shows all characteristic peaks for quartz (SiO_2_) and calcite (CaCO_3_), consistent with mineral phases expected in argillaceous limestone. The reference spectra were downloaded from the RRUFF database (#040031 and #040070).

**
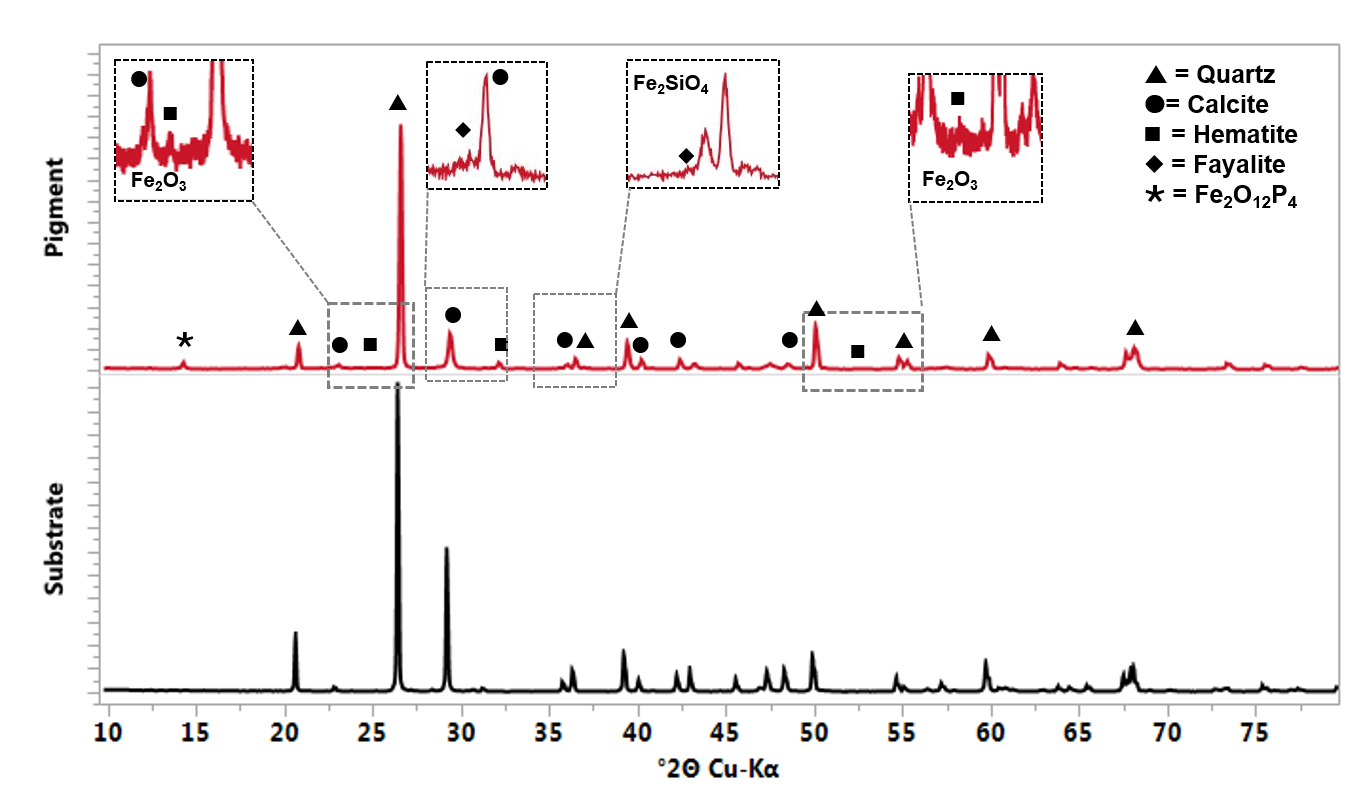
**

**SI Figure 5: Comparison of XRD spectra GcSi-1 painted side and rock substrate.** Quartz and calcite are dominant phases in both spectra. Although low in abundance hematite, fayalite, and iron phosphate were detected in the paint layer. Reference spectra used for comparison were downloaded from the RRUFF database: quartz (#040031) calcite (#040070), hematite (#R040024), and fayalite (#R070157).

**
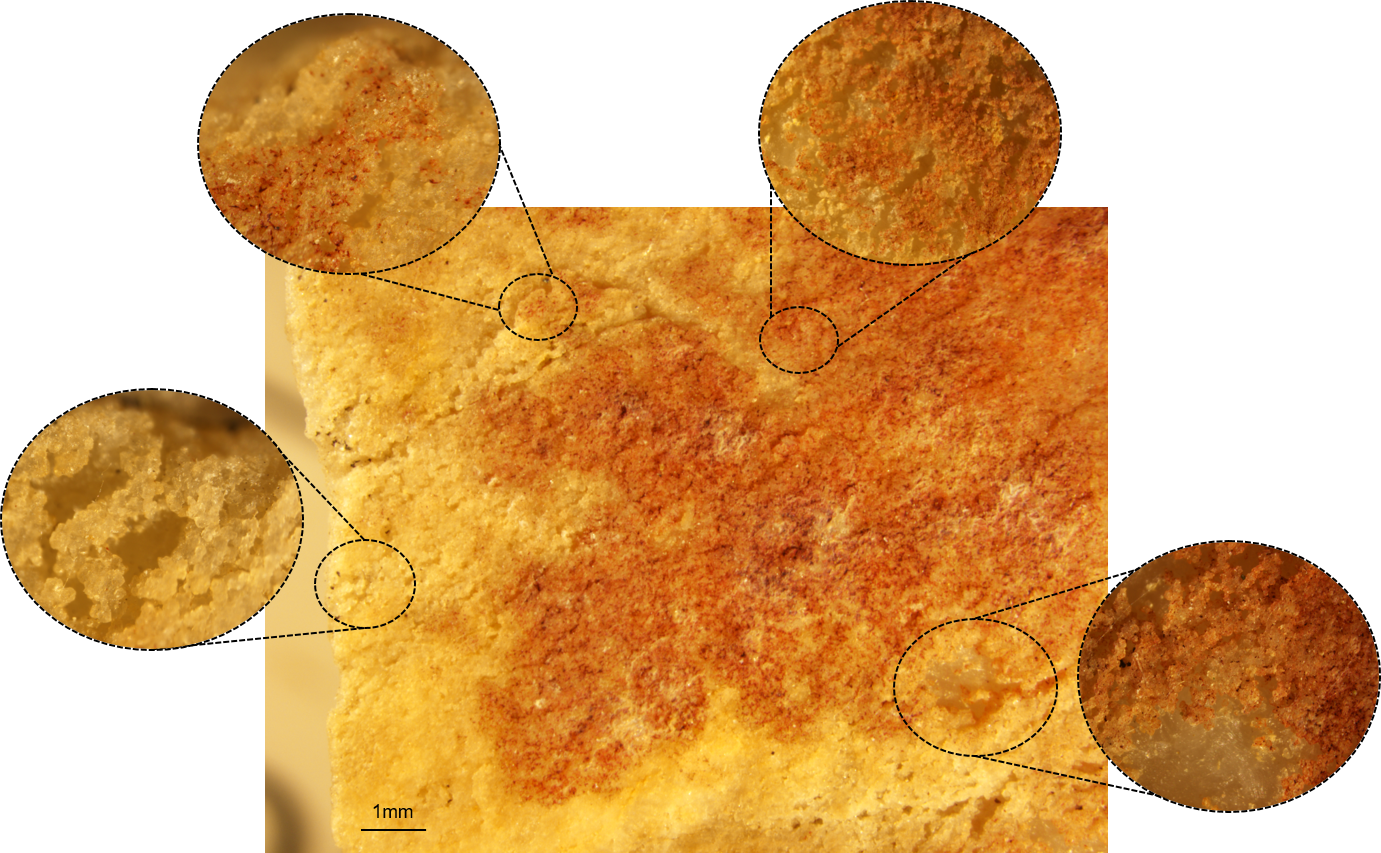
**

**SI Figure 6: Low magnification optical micrographs of GcSi-1.** Details of rock face surface texture are shown at low magnification with low raking light to enhance micro-topography. A layer of crystalline calcite growth overlies the silicified limestone substrate. Red pigment particles are embedded in subsequently and overlain by the calcite formation.


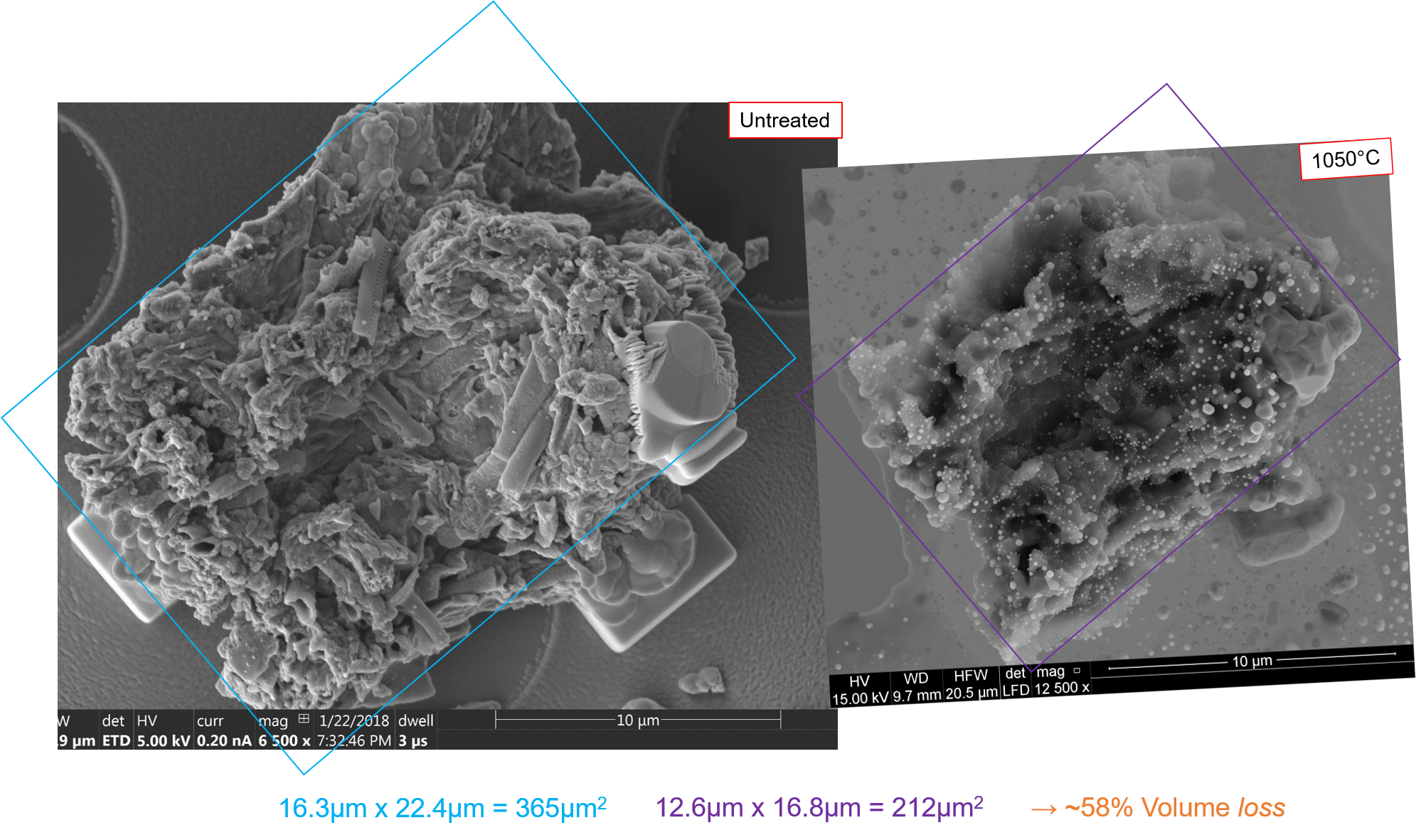


**SI Figure 7: Comparison of FeOB particle before and during *in situ* thermal experiment**. *Left:* the untreated pigment particle; and; *Right:* heated to 1,050°C, illustrating an approximate 58% volume loss during the heating procedure. Note the proliferation and concentration of hematite microspheres in the treated sample.


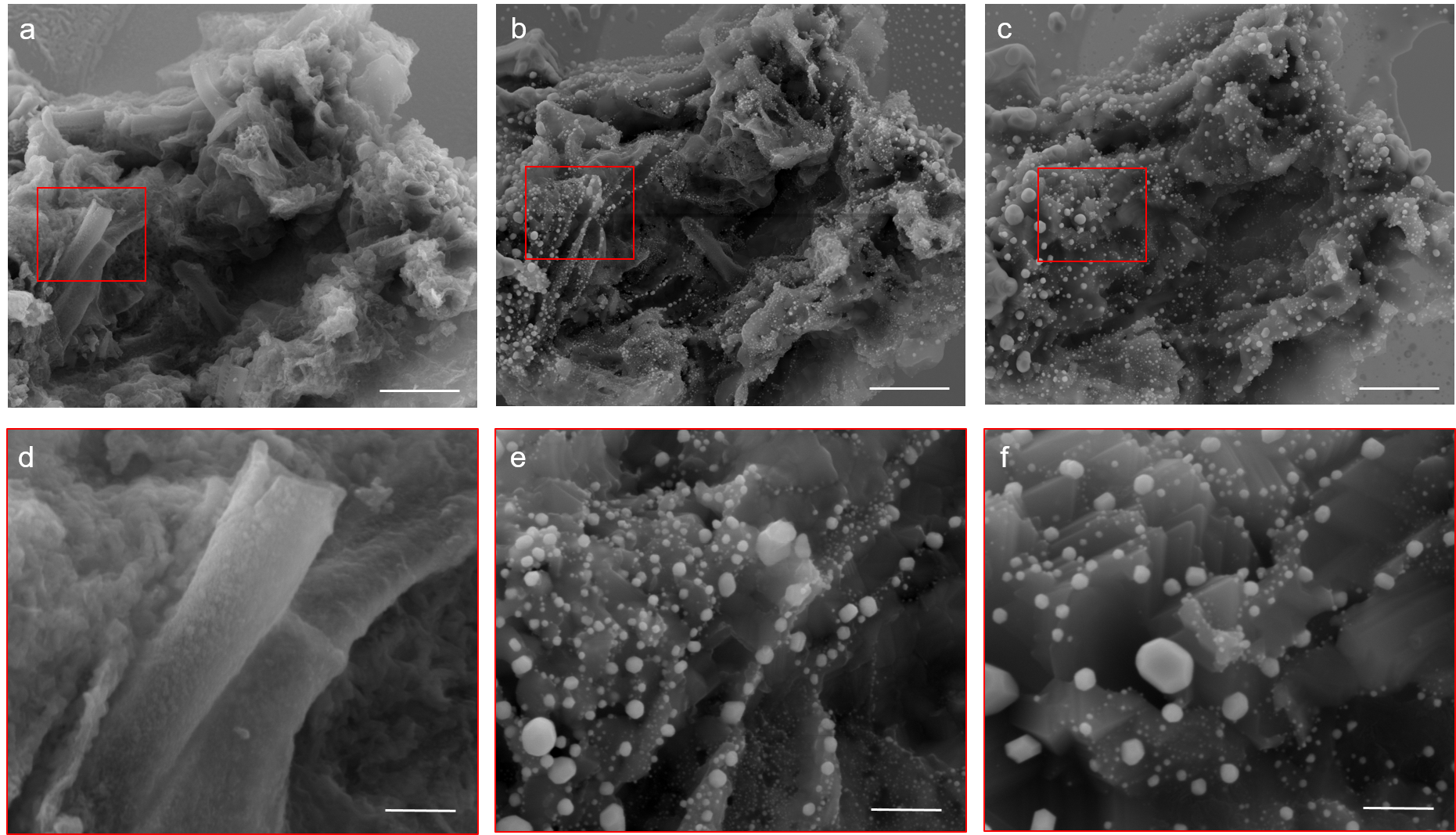


**SI Figure 8: SEM micrographs of *L. ochracea* at 600°C, 950°C, and 1050°C during *in situ* heating**. Note the lack of melt-phase separation at 600°C (a, d), with increasing structural degradation at 950°C (b, e) and 1050°C (c, f). Below 950°C the hematite and magnetite microspheres began to coalesce into larger particles. The *L. ochracea* sheath structure was still minimally visible though rapidly degrading. At 1050°C, the formation of angular hematite and magnetite polymorphs proliferated. Scales a, b, c = 2.5 µm. Scales d, e, f = 0.5 µm.


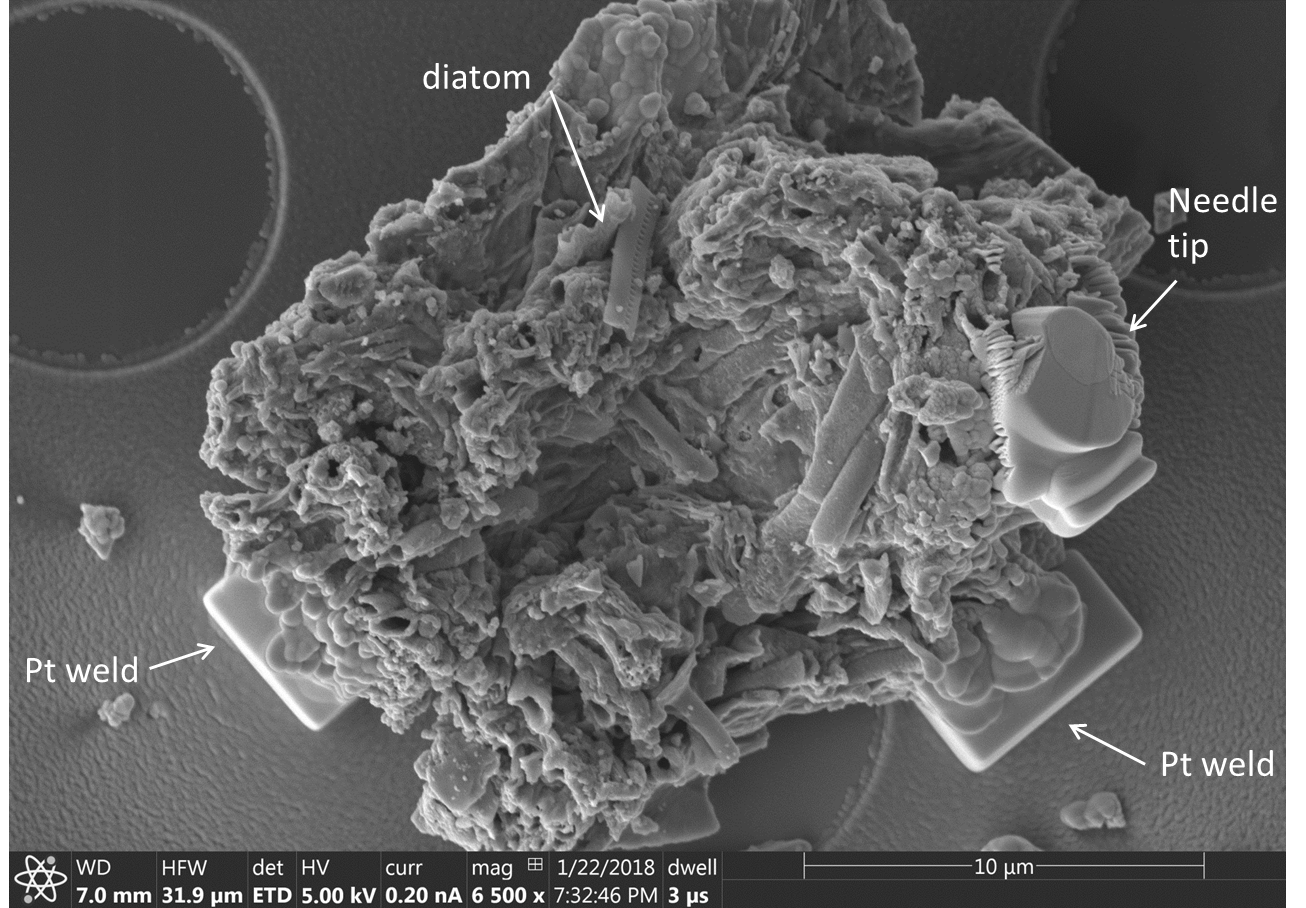


**SI Figure 9: Untreated FeOB control sample prepared for *in situ* SEM heating experiment.** Sample was affixed to the Protochips Fusion microchip using FIB-SEM.

**
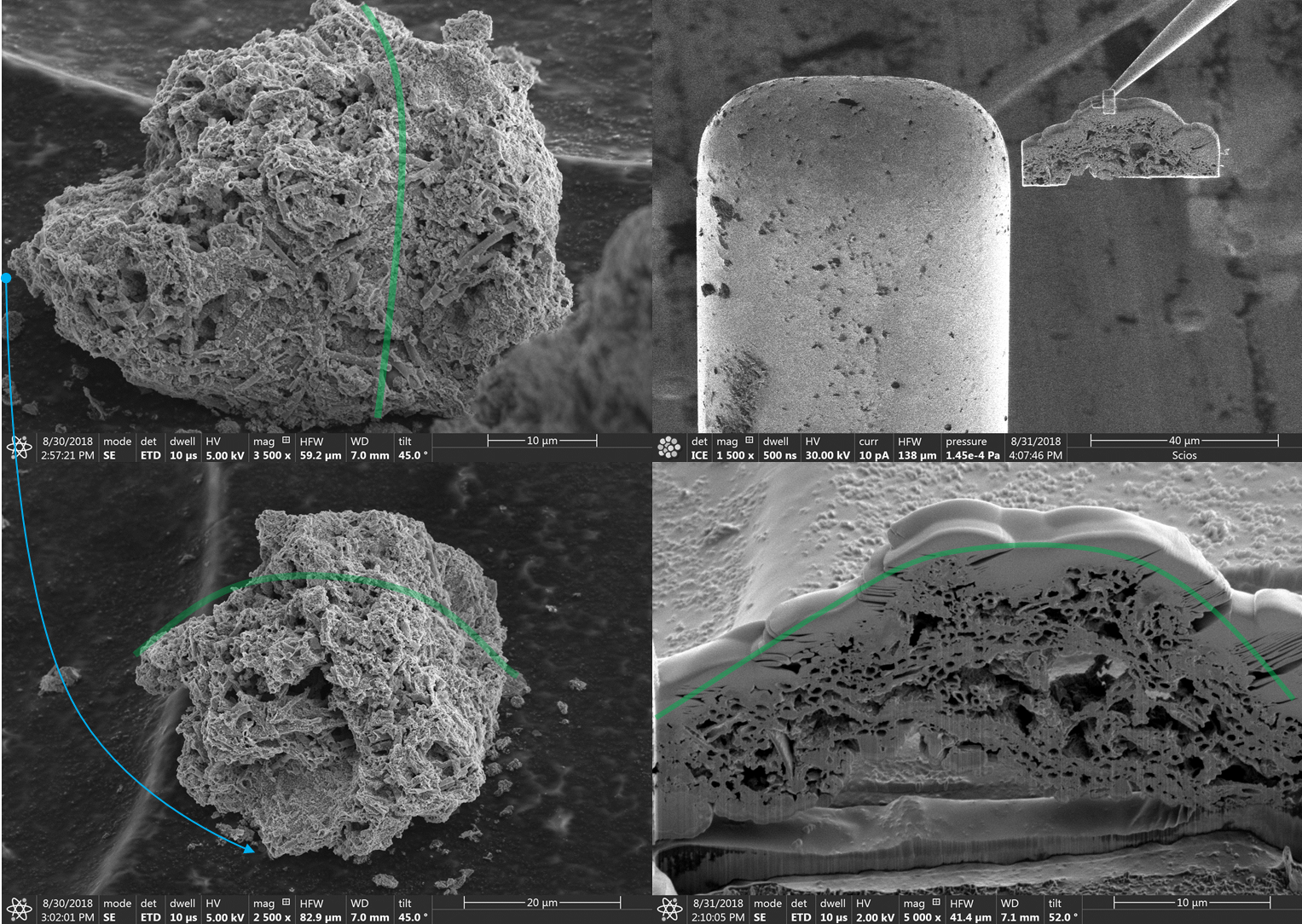
**

**SI Figure 10: Series of micrographs showing the use of FIB-SEM to prepare FeOB 800°C control sample for HRTEM analysis.** The sediment mass was ablated with a focused Ga ion beam to expose a 2 µm thin cross section of a cluster of *L. ochracea* sheaths. The cross section was lifted out and polished in preparation for HRTEM.


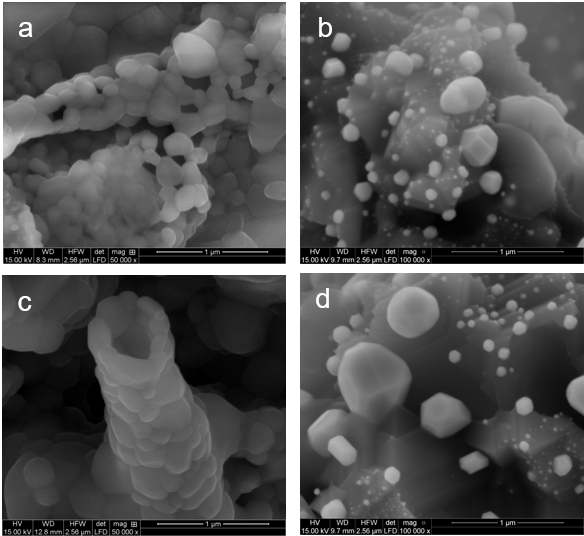


**SI Figure 11: Details of *L. ochracea* sheath morphology.** (a, c) muffle furnace heated samples (1,000°C). *L. ochracea* sheath structures are highly transformed, showing nucleation of highly-crystalline iron oxide polymorphs on exterior surfaces. (b, d) sample heated to 1,050°C using *in situ* SEM heating. The *L. ochracea* sheaths are fully degraded and iron oxide nanoparticles form and coalesce as temperature increases.


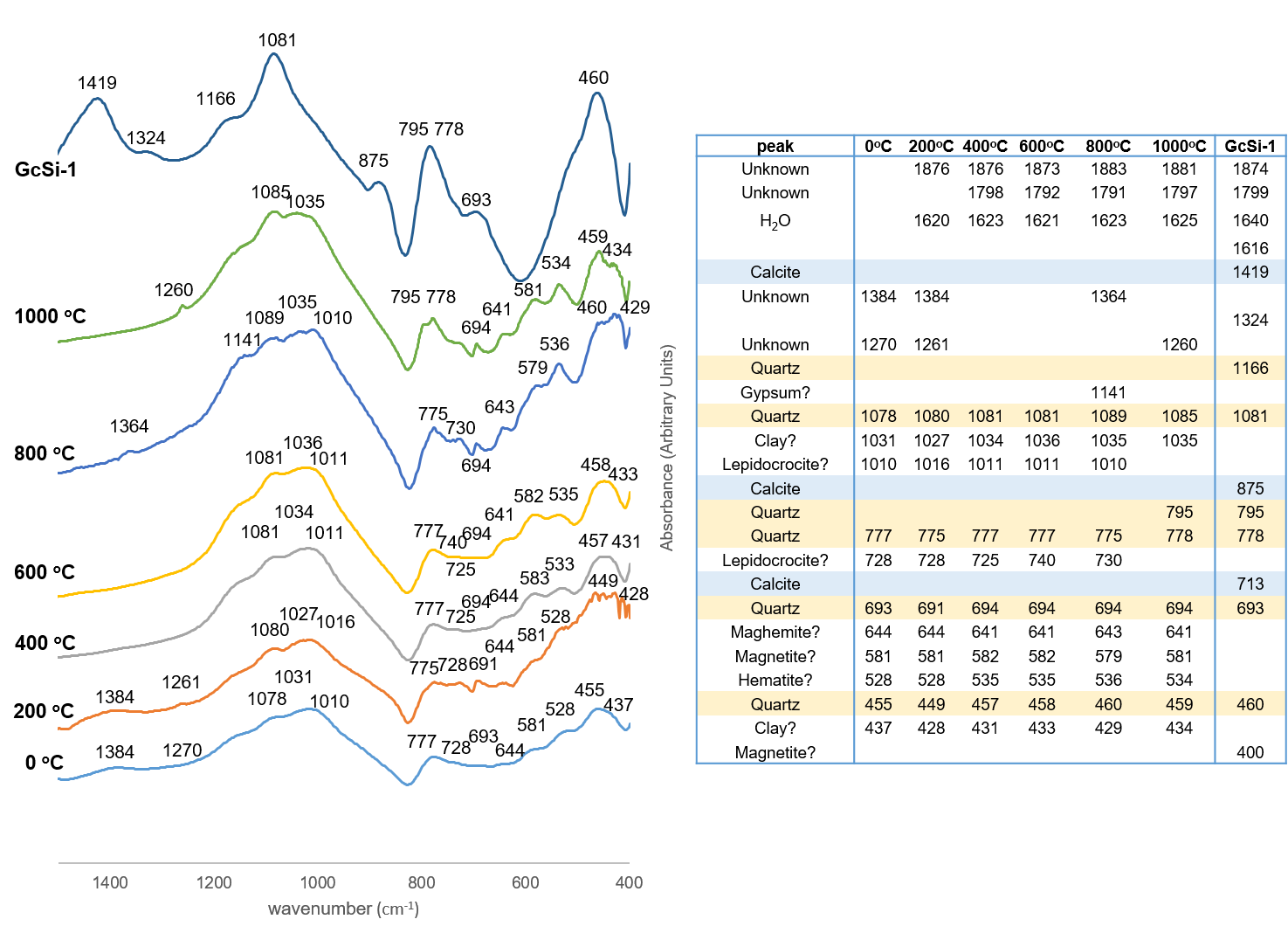


**SI Figure 12: ATR-FTIR spectra of FeOB control samples and GcSi-1.** Major peaks for calcite and quartz are readily identifiable, however most iron oxides are difficult to resolve or confirm due to spectral interferences. Only data for wavenumbers 400 cm^-1^ – 1400 cm^-1^ are shown. Wavenumber references were taken from Namduri and Nasrazadani 2008 [^34^](#_ENREF_34).

**Supplementary Table 1: Summary of mineral phases identified in GcSi-1 and heat treated FeOB control samples.** *Phases confirmed by HRTEM. All others identified by powder XRD.

| **Phase Type** | **BP Paint** | **BP Rock** | **FeOB 0°C** | **FeOB 200°C** | **FeOB 400°C** | **FeOB 600°C** | **FeOB 800°C** | **FeOB 1,000°C** |
| --- | --- | --- | --- | --- | --- | --- | --- | --- |
| **Iron Oxides** | | | | | | | | |
| **Iron phosphate oxide/hydroxide**  Fe_4_PO, FeHO_5_P, Fe_3_O_8_P_2,_ Fe_3_(PO_4_)_2_O(OH) | **?** |  | **X** | **X** | **X** | **X** |  | **X** |
| **Iron oxide/oxyhydroxide,**  FeO; FeO(OH), FeO(OH)·*n*H_2_O, (Fe,Mg)O |  |  | **X** | **X** | **X** | **X** | **X** | **X** |
| **Lepidocrocite** FeO_2_ |  |  | **?** |  | **X** |  |  |  |
| **Iron silicate** FeSi, Fe_2_SiO_4_ | **X** |  |  | **?** |  |  | **X** | **X** |
| **Ferrihydrite/ hydrous ferrous** **oxyhydroxide** 5Fe_2_O_3_•9H_2_O |  |  |  |  | **X** |  |  |  |
| **Hematite** Fe_2_O_3_ | **X*** |  |  |  |  | **X** | **X*** |  |
| **Magnetite**  Fe_3_O_4_ | **X*** |  |  |  | **X** | **X** | **X*** | **X** |
| **Maghemite** γ-Fe_2_O_3_ | **X*** |  |  |  |  |  |  |  |
| **Other** | | | | | | | | |
| **Quartz** SiO_2_ | **X** | **X** | **X** | **X** | **X** | **X** | **X** | **X** |
| **Cristobalite** SiO |  |  |  |  |  | **X** | **X** | **X** |
| **Calcite** CaCO_3_ | **X** | **X** |  |  |  |  |  |  |
| **FeS** |  |  |  |  |  | **X** |  |  |
| **Carbon (charred organic)** |  |  |  | **?** |  | **?** |  | **?** |
| **Ankerite** Ca(Fe,Mg)(CO_3_)_2_ |  |  | **X** | **X** | **X** | **X** |  |  |
| **Almandine** Fe_3_Al_2_Si_3_O_12_ |  |  |  |  | **X** | **X** | **X** |  |

References Cited

1 Arnett, C. A. *Rock art of Nlaka'pamux: indigenous theory and practice on the British Columbia Plateau*, University of British Columbia, (2016).

2 Corner, J. *Pictographs (Indian rock paintings) in the Interior of British Columbia*. (Wayside Press, 1968).

3 Wainwright, I. N. Rock art conservation research in Canada. *Bollettino del Centro Camuno di studi preistorici* **22**, 15-46 (1985).

4 Arnett, C. & Morin, J. The Rock Painting/Xela: ls of the Tsleil-Waututh: A Historicized Coast Salish Practice. *Ethnohistory* **65**, 101-127, doi:10.1215/00141801-4260674 (2018).

5 Carlson, R. L. in *Time and space: dating and spatial considerations in rock art research* 7-12 (1993).

6 Williams, J. *Two Wolves at the Dawn of Time: Kingcome Inlet pictographs, 1983-1998*. (New Star Books, 2001).

7 York, A., R. Daly, and C. Arnett. *They write their dreams on the rock forever: rock writings of the Stein River Valley of British Columbia*. (Talonbooks, 1993).

8 Ames, K. M. Report of Excavations at GhSv 2, Hagwilget Canyon. *GF MacDonald and RI Inglis, Mercury Series, Archaeological Survey Papers* **87** (1979).

9 Mohs, A., and G. Mohs. Babine Lake Archaeological Survey Project, 1976-5. (British Columbia Heritage Conservation Branch, Victoria, British Columbia, Canada, 1976).

10 Bishop, C. A. Coast-interior exchange: the origins of stratification in Northwestern North America. *Arctic Anthropology*, 72-83 (1987).

11 Morice, A. G. *The History of the Northern Interior of British Columbia 1660-1880*. (Ye Gallon Press, 1906).

12 Reimer, R. *The Mountains and Rocks are Forever: Lithics and Landscapes of skwxwú7mesh uxwumixw* PhD thesis, McMaster University, (2012).

13 Rahemtulla, F. Archaeological Research Investigations at Site GiSq-004 Located at Nilitkwa Lake in the North-Central Interior of BC. *Report on File with the British Columbia Archaeology Branch, Victoria* (2012).

14 Rahemtulla, F. Archaeological Research Investigations at Site GiSq-004 Located at Nilitkwa Lake in the North-Central Interior of BC. *Report on File with the British Columbia Archaeology Branch, Victoria* (2013

).

15 Mitchell, D. & Donald, L. Archaeology and the study of Northwest Coast economies. *Prehistoric Economies of the Pacific Northwest Coast, Research in Economic Anthropology Supplement* **3**, 293-351 (1988).

16 Rahemtulla, F. in *Waterlogged: Emerging Trends in Northwest Coast Archaeology* (ed Kathryn Bernick) 159-168 (Washington State University Press, 2019).

17 Schiarizza, P. & MacIntyre, D. Geology of the Babine Lake–Takla Lake area, central British Columbia (93 K/11, 12, 13, 14; 93 N/3, 4, 5, 6). *Geological fieldwork*, 1999-1991 (1998).

18 Kappler, A. & Newman, D. K. Formation of Fe (III)-minerals by Fe (II)-oxidizing photoautotrophic bacteria. *Geochimica et Cosmochimica Acta* **68**, 1217-1226 (2004).

19 Edwards, K. J., Bach, W., McCollom, T. M. & Rogers, D. R. Neutrophilic iron-oxidizing bacteria in the ocean: their habitats, diversity, and roles in mineral deposition, rock alteration, and biomass production in the deep-sea. *Geomicrobiology Journal* **21**, 393-404 (2004).

20 Konhauser, K. O., Kappler, A. & Roden, E. E. Iron in microbial metabolisms. *Elements* **7**, 89-93 (2011).

21 Parenteau, M. N. & Cady, S. L. Microbial biosignatures in iron-mineralized phototrophic mats at Chocolate Pots hot springs, Yellowstone National Park, United States. *Palaios* **25**, 97-111 (2010).

22 Vargas, M., Kashefi, K., Blunt-Harris, E. L. & Lovley, D. R. Microbiological evidence for Fe (III) reduction on early Earth. *Nature* **395**, 65 (1998).

23 Kunoh, T., Kunoh, H. & Takada, J. Perspectives on the biogenesis of iron oxide complexes produced by Leptothrix, an iron-oxidizing bacterium and promising industrial applications for their functions. *J. Microb. Biochem. Technol.* **7**, 419-426 (2015).

24 Fleming, E. *et al.* Insights into the fundamental physiology of the uncultured Fe-oxidizing bacterium Leptothrix ochracea. *Appl. Environ. Microbiol.* **84**, e02239-02217 (2018).

25 Fortin, D. & Langley, S. Formation and occurrence of biogenic iron-rich minerals. *Earth-Science Reviews* **72**, 1-19 (2005).

26 Emerson, D. & Weiss, J. V. Bacterial iron oxidation in circumneutral freshwater habitats: findings from the field and the laboratory. *Geomicrobiology Journal* **21**, 405-414 (2004).

27 Posth, N. R. *et al.* Simulating Precambrian banded iron formation diagenesis. *Chemical Geology* **362**, 66-73 (2013).

28 Chan, C. S. *et al.* The architecture of iron microbial mats reflects the adaptation of chemolithotrophic iron oxidation in freshwater and marine environments. *Frontiers in microbiology* **7**, 796 (2016).

29 Angelova, R. *et al.* Biogenic iron oxides from laboratory cultivated Leptothrix sp. for application in the bionanotechnology. *Annuaire de l’Université de Sofia*, 231-238 (2015).

30 Suzuki, T. *et al.* Structural and spatial associations between Fe, O, and C in the network structure of the Leptothrix ochracea sheath surface. *Appl. Environ. Microbiol.* **77**, 7873-7875 (2011).

31 Birle, J., Gibbs, G., Moore, P. & Smith, J. Crystal structures of natural olivines. *American Mineralogist: Journal of Earth and Planetary Materials* **53**, 807-824 (1968).

32 Yuan, Q., Xu, G., Zhou, M., He, B. & Hu, H. The effect of p on the microstructure and melting temperature of Fe2SiO4 in silicon-containing steels investigated by in situ observation. *Metals* **7**, 37 (2017).

33 Tsatskin, A. & Gendler, T. S. Identification of “red ochre” in soil at Kfar HaHoresh Neolithic site, Israel: Magnetic measurements coupled with materials characterization. *Journal of Archaeological Science: Reports* **6**, 284-292 (2016).

34 Namduri, H. & Nasrazadani, S. Quantitative analysis of iron oxides using Fourier transform infrared spectrophotometry. *Corrosion Science* **50**, 2493-2497 (2008).

35 Chio, C. H., Sharma, S. K. & Muenow, D. W. The hydrates and deuterates of ferrous sulfate (FeSO4): a Raman spectroscopic study. *Journal of Raman Spectroscopy: An International Journal for Original Work in all Aspects of Raman Spectroscopy, Including Higher Order Processes, and also Brillouin and Rayleigh Scattering* **38**, 87-99 (2007).
